# circHIPK3 prevents cardiac senescence by acting as a scaffold to recruit ubiquitin ligase to degrade HuR

**DOI:** 10.7150/thno.77630

**Published:** 2022-10-31

**Authors:** Fengzhi Ding, Lin Lu, Chengjie Wu, Xiangbin Pan, Bin Liu, Yu Zhang, Yanli Wang, Weiliang Wu, Bing Yan, Yuqing Zhang, Xi-Yong Yu, Yangxin Li

**Affiliations:** 1 Institute for Cardiovascular Science and Department of Cardiovascular Surgery, First Affiliated Hospital and Medical College of Soochow University, Collaborative Innovation Center of Hematology, Soochow University, Suzhou, Jiangsu 215123, P. R. China.; 2 Department of Structural Heart Disease, National Center for Cardiovascular Disease, China & Fuwai Hospital, Chinese Academy of Medical Sciences & Peking Union Medical College, Beijing, Key Laboratory of Cardiovascular Appratus Innovation, Beijing 100037, P.R. China.; 3 Department of Cardiology, the Second Hospital of Jilin University, Changchun, Jilin 130041, P. R. China.; 4 Key Laboratory of Molecular Target & Clinical Pharmacology and the NMPA & State Key Laboratory of Respiratory Disease, Guangzhou Medical University, Guangzhou, Guangdong 511436, P. R. China.

**Keywords:** exosome, circHIPK3, RNA-binding protein, senescence, ubiquitin ligase, aging

## Abstract

**Rational:** Senescence is a major aging process that contributes to the development of cardiovascular diseases, but the underlying molecular mechanisms remain largely unknown. One reason is due to the lack of suitable animal models. We aimed to generate a cardiomyocyte (CM)-specific senescent animal model, uncover the underlying mechanisms, and develop new therapies for aging associated cardiac dysfunction.

**Methods:** The gain/loss of circHIPK3 approach was used to explore the role of circHIPK3 in cardiomyocyte (CM) senescence. To investigate the mechanisms of circHIPK3 function in cardiac senescence, we generated CM-specific tamoxifen-induced circHIPK3 knockout (CKO) mice. We also applied various analyses including PCR, Western blot, nuclear and cytoplasmic protein extraction, immunofluorescence, echocardiography, RNA immunoprecipitation assay, RNA-pulldown assay, and co-immunoprecipitation.

**Results:** Our novel CKO mice exhibited worse cardiac function, decreased circHIPK3 expression and telomere length shortening in the heart. The level of the senescence-inducer p21 in the hearts of CKO mice was significantly increased and survival was poor compared with control mice. *In vitro*, the level of p21 in CMs was significantly decreased by circHIPK3 overexpression, but increased by circHIPK3 silencing. We showed that circHIPK3 was a scaffold for p21 mRNA-binding protein HuR and E3 ubiquitin ligase β-TrCP. circHIPK3 silencing weakened the interaction between HuR and β-TrCP, reduced HuR ubiquitination, and enhanced the interaction between HuR and p21 mRNA. Moreover, we found that mice injected with human umbilical cord mesenchymal stem cell-derived exosomes (UMSC-Exos) showed increased circHIPK3 levels, decreased levels of p21, longer telomere length, and good cardiac function. However, these beneficial effects exerted by UMSC-Exos were inhibited by silencing circHIPK3.

**Conclusions:** We successfully generated CM-specific CKO mice for aging research. Our results showed that deletion of circHIPK3 led to exaggerated CM senescence and decreased cardiac function. As a scaffold, circHIPK3 enhanced the binding of E3 ubiquitin ligase β-TrCP and HuR in the cytoplasm, leading to the ubiquitination and degradation of HuR and reduced p21 activity. In addition, UMSC-Exos exerted an anti-senescence and cardio-protective effect by delivering circHIPK3. These findings pave the way to the development of new therapies for aging associated cardiac dysfunction.

## Introduction

The incidence of cardiovascular diseases such as myocardial infarction and heart failure increases with aging 1. Senescence is a major aging process whereby increasing numbers of cells undergo permanent growth arrest. The accumulation of senescent cells eventually leads to tissue damage and aging-related diseases 2. Senescence of cardiomyocytes (CMs) is characterized by increased expression of p21 and telomere shortening 3. Still, the molecular mechanisms responsible for senescence remain largely unknown due to the lack of suitable animal models that mimic the human aging process. Currently, there is no effective treatment to prevent or delay cardiac senescence.

Circular RNAs (circRNAs) are a class of single-stranded RNAs that form a closed loop with regulatory functions 4. Previous studies have shown that the expression of circSfl is increased in long-lived flies, and the life span of regular flies can be prolonged by overexpressing circSfl 5. Garikipati *et al*. showed that the expression level of circHIPK3 is significantly lower in the infarcted hearts than in the controls, suggesting that circHIPK3 may regulate cardiac function 6. Moreover, previous studies have shown that circHIPK3 promotes angiogenesis in infarcted myocardium 7, and this beneficial effect is mediated by circHIPK3 which acts as a sponge for miR-133a 8. Others have also discovered that circHIPK3 enhances cell proliferation, and decreased proliferation is a hallmark of senescence 9. Recently, we found that circHIPK3 promotes recovery of the ischemic hind limb in mice 10. Importantly, our novel circRNA sequencing data from young and aging hearts revealed that circHIPK3 is one of the most abundant circRNAs in the heart and its expression decreases dramatically during aging. These studies suggest that senescence is regulated by circRNA, but its effect on cardiac senescence has not been explored. In this study, we generated inducible CM-specific circHIPK3 knockout (CKO) mice to explore the impact of circHIPK3 deletion on cardiac senescence and uncover the underlying mechanisms.

Human antigen R (HuR) is an RNA-binding protein that binds to the AU-rich element in the 3' untranslated region of specific mRNAs, including those involved in cell growth, apoptosis, and survival by modulating the stability of the target mRNA. HuR is primarily localized in the nucleus but is translocated into the cytoplasm upon ischemic injury 11. The relocation of HuR is responsible for impaired cardiac function 11. These studies suggest that the cellular location of HuR is linked to its function. It has been shown that HuR enhances the expression of the senescence inducer p21 12, 13. However, the role of HuR in cardiac senescence has not been established, and the link between circHIPK3 and HuR remains unexplored.

Previous studies have shown that HuR can be degraded by the ubiquitin-proteasome system (UPS) 14 through the interaction with E3 ubiquitin ligase β-transducin repeat-containing protein (β-TrCP) 15. The UPS maintains cell functions by eliminating dysfunctional, misfolded, or damaged proteins through the selective ubiquitination of target proteins that are broken down into small molecules in the proteasome. Protein ubiquitination is a three-step process involving the ubiquitin-activating enzyme E1, conjugating enzyme E2, and ligase E3. Among these enzymes, E3 ligase is most critical because it recognizes specific substrate proteins for degradation 16. β-TrCP is one of the E3 ligases that targets IκB for degradation 17, 18. However, the link between circHIPK3, HuR, and β-TrCP has not been explored.

Exosomes are cell-derived microvesicles containing noncoding RNAs, including microRNAs, lncRNAs, and circRNAs 19, 20. Our previous studies have demonstrated that human umbilical cord mesenchymal stem cell-derived exosomes (UMSC-Exos) inhibit aging-induced vascular dysfunction and cardiac dysfunction by releasing miR-675 21, and the lncRNA MALAT1 22. However, the molecular mechanisms underlying the anti-senescence effect of UMSC-Exos remain largely unknown. Moreover, due to the instability of microRNAs and the poor conservation of lncRNAs, the clinical application of microRNAs and lncRNAs as therapeutic agents is limited. Recently, most research has focused on circRNAs resistant to exonuclease-mediated degradation and conserved in vertebrates.

This study generated novel CM-specific, tamoxifen-induced circHIPK3-CKO mice for aging research. Using discovery-driven approaches, including circRNA/mRNA sequencing, RIP assay, and RNA-pulldown assay, we found that circHIPK3 serves as scaffold for HuR and the ubiquitin E3 ligase β-TrCP to promote HuR ubiquitination and degradation. Therefore, circHIPK3 reduces cardiac senescence and prevents cardiac dysfunction by clearing HuR and reducing p21 activity. Furthermore, we found that UMSC-Exos can restore cardiac function in circHIPK3-CKO mice by releasing circHIPK3 to inhibit cardiac senescence.

## Methods

The data, methods and materials related to this study are available to other researchers on reasonable request.

### Generation of circHIPK3 knockout mice

To generate circHIPK3 knockout (KO) mice, a construct was engineered for the disruption of the circHIPK3, with two loxP sites flanking the downstream of short interspersed elements (SINEs) sequence of exon 2 of the HIPK3 gene. To engineer the targeting vector, homology arms and knockout region were generated by PCR using Bacterial Artificial Chromosome (BAC) clone RP24-242C16 and RP24-245G14 from the C57BL/6 library as template. In the targeting vector, the Neo cassette was flanked by SDA (self-deletion anchor) sites. After the vector was linearized, ES cells were transfected by electroporation. Positive clones were selected by G418 treatment and verified by long-fragment PCR identification. Diphtheria toxin (DTA) was used for negative selection. Positive clones with correct homologous recombination were expanded and injected into blastocysts of C57BL/6J mice to obtain chimeric mice (circHIPK3^Flox/WT^). The circHIPK3^Flox/WT^ mice were generated by the Cyagen Biosciences Inc. The genotype of circHIPK3^Flox/Flox^ mouse was confirmed by Sanger sequencing.

αMHC-driving CM-specific knockout mice were generated by crossing αMHC^Cre/WT^ mice with circHIPK3^Flox/Flox^ mice. The heterozygous αMHC^Cre/WT^/circHIPK3^Flox/WT^ mice were back-crossed with the circHIPK3^Flox/Flox^ mice to obtain CM-specific circHIPK3 KO mice. The mouse genotype was identified by One Step Mouse Genotyping Kit (Vazyme, China). The KO mice exhibited early cardiac senescence and cardiac dysfunction and the reproduction rate was very slow, suggesting that deletion of circHIPK3 impaired development. To overcome this problem, we generated inducible CM-specific circHIPK3 knockout (CKO) mice by crossing αMHC^MerCreMer^ mice with circHIPK3^Flox/Flox^ mice. Induction of Cre recombinase activity was achieved using tamoxifen (Sigma, 100 μg/g body weight) dissolved in corn oil (Solarbio, China) and administrated intraperitoneally (i.p.). After tamoxifen administration, the hearts of CKO mice were harvested to confirm the deletion of circHIPK3.

All animal procedures were approved by the Institutional Animal Care and Use Committee at Soochow University (Suzhou, China). All animal experiments comply with the ARRIVE guidelines and are carried out in accordance with the National Institutes of Health guide for the care and use of Laboratory animals (NIH Publications No. 8023, revised 1978) and the manuscript has followed such guidelines.

### circRNA/mRNA sequencing and analysis

Young (6 weeks) and middle-aged (15 months) male C57BL/6 mice were used for circRNA/mRNA sequencing and analysis. Total RNA was extracted from the hearts of the mice by Trizol reagent (Invitrogen). Ribosome depleted RNA samples were fragmented and then used for first- and second-strand complementary DNA (cDNA) synthesis with random hexamer primers. Whole transcriptome sequencing data obtained from HiseqTM Sequencer was filtered (removing the adaptor sequences, reads with > 5% ambiguous bases and low-quality reads containing more than 20 percent of bases with qualities of < 20) and mapped to mouse genome utilizing HISAT2. HTSeq was used to calculate the gene count of mRNA and circRNA. All RNA-seq and bioinformatic analysis were performed at NovelBio Ltd (Shanghai, China).

### Isolation of mouse cardiomyocytes (CMs)

Primary CMs were isolated from neonatal C57BL/6 mice as described previously 23. Briefly, the hearts were cut into small pieces (1-3 mm^2^), digested by 0.25% trypsin at 37 °C, and the supernatant was collected into Dulbecco's modified Eagle's medium (DMEM) containing with 10% fetal bovine serum. After the tissue was completely digested, the supernatant was filtered through a cell strainer (100 μm) and then centrifuged at 1000 rpm for 5 min. The pellets were resuspended in DMEM containing with 10% fetal bovine serum. The cells were seeded onto 10 cm plastic dishes for 2 h at 37 °C to remove fibroblasts, and then plated on 1% gelatin-coated plastic culture dishes.

### Evaluation of cardiac function

Echocardiography was performed to evaluate cardiac function using a 13 MHz transducer (VisualSonics). The left ventricular ejection fraction (EF) and fraction shorting (FS) were calculated. All procedures and analysis were performed by a researcher who was blinded to treatment groups.

### Harvest and identification of UMSC exosomes

Human umbilical mesenchymal stem cells (UMSCs; Jiangsu Heze Biotechnology Co., Ltd., China) were cultured in minimum essential medium (MEM) with 10% fetal bovine serum (FBS). The FBS had been centrifuged at 100,000 g to eliminate preexisting bovine-derived exosomes. After 48 h in culture, exosomes were isolated from UMSC culture supernatant using a total exosome isolation kit (Life Technology, Grand Island, NY, USA). The culture medium collected from UMSCs was centrifuged at 2,000 g for 30 min to remove dead cells and debris, and then transferred to a new tube containing 0.5 volumes of the Total Exosome Isolation reagent. The mixture was incubated at 4 °C overnight and centrifuged at 10,000 g for 1 h at 4 °C. The pellets (exosomes) were resuspended in phosphate buffer saline. The concentration of exosomes was determined using a bicinchoninic acid (BCA) protein assay kit (Takara, Japan). The exosomes were attached to aldehyde/sulphate latex beads (4 μm, Molecular Probes, Invitrogen), then incubated with a FITC‐conjugated antibody against CD63 (Abcam), and the expression of CD63 was analyzed by flow cytometry. In separate experiments, the expression of exosome marker CD9 was also analyzed by Western blot.

### Plasmid and siRNA construction

Small interference RNA targeting circHIPK3 (circHIPK3 siRNA) was synthesized by Ribobio (Guangzhou, China). For experiments involving si-Exo, the UMSCs were transfected with circHIPK3 siRNA for 40 h, and then the exosomes were isolated, as mentioned above.

The sequence of circHIPK3 was amplified and cloned into a circRNA overexpression vector pK5ssAAV-ciR (Geneseed, Guangzhou, China) through restriction enzyme sites EcoRI and BamHI, and confirmed by sequencing. The empty vector was used as negative control. After transfection for 40 h, H9C2 cells were harvested for subsequent experiments. siRNA sequence is listed in Table S1 in the online-only Data Supplement. Cells were transfected with plasmid or siRNA using Lipofectamine 2000 (Invitrogen, Carlsbad, CA, USA) according to the manufacturer's instructions.

### Exosome labeling with PKH26

Purified exosomes were labeled with PKH26 fluorescent labeling kit (Sigma-Aldrich, St Louis, USA). H9C2 cells were grown to 50% confluence in 12-well plates, and then the medium was replaced with DMEM containing PKH26-labeled exosomes. After incubation for 24 h at 37 °C in 5% CO_2_ atmosphere, the cells were washed twice with PBS, fixed, and nuclei were stained with 4′, 6-diamidino-2-phenylindole (DAPI). Finally, the sample of the cells was determined with fluorescence microscopy.

### Nanoparticle size analysis

Exosome size and number of particles were assessed using a NanoSight NS300 equipped with a 405 nm laser (Malvern, Great Malvern, UK). Videos (60 s duration, 30 frames/sec) were recorded and particle movement was analyzed using the NTA software (NanoSight version 2.3).

### EdU proliferation assay

Cell proliferation was assessed using EdU Cell Proliferation Assay kit (RiboBio, Guangzhou, China). After different treatments, primary cardiomyocytes were incubated in fresh medium containing 10 μM EdU for 24 h, then cardiomyocytes were washed with PBS, and fixed with 4% paraformaldehyde for 30 min, treated with 0.5% Triton X-100 for 10 min. Cell nuclei were stained with DAPI for 15 minutes. Finally, the proportion of the cells incorporating EdU was determined with fluorescence microscopy.

### Senescence-associated β-galactosidase (β-gal) staining

The β-gal staining was performed using senescence β-galactosidase staining kit (Beyotime Biotechnology, China) per manufacturer's instructions. In brief, neonatal primary cardiomyocytes were fixed at room temperature for 15 min, washed in PBS and stained in β-galactosidase solution at 37 °C overnight. The number of positive cells was counted under a light microscope.

### RNA extraction and real-time PCR

RNA was extracted using Trizol reagent (TaKaRa Biotech, Japan), and cDNA was synthesized using Prime Script™ RT reagent (TaKaRa Biotech). The relative expression levels of target genes were determined using SYBR® Premix Ex Taq™ (TliRNaseH Plus) (TaKaRa Biotech). Real-time PCR was performed using the ABI (Foster City, CA, USA) StepOnePlus Real-Time PCR System. The primers used were listed in Table S1 in the Data Supplement.

### Western blot

Total proteins extracted by RIPA buffer (Beyotime Biotechnology, China) were separated by SDS-polyacrylamide gel electrophoresis (SDS-PAGE), and then transferred to polyvinylidene fluoride (PVDF) membranes (Millipore ISEQ00010). The membranes were first incubated with primary antibodies followed by secondary antibodies. The primary antibodies against p16 (Cat#32050) and p21 (Cat#30427) were from Signalway Antibody (Baltimore, MD, USA). Antibody against GAPDH (Cat#60004-1-lg) was from Proteintech (Rosemount, IL, USA). Antibodies against HuR (Cat#sc-5261) and CD63 (Cat#sc-5275) were obtain from Santa Cruz Biotechnology (Dallas, TX, USA). The primary antibodies against CD9 (Cat#ab92726), were from Abcam (Cambridge, MA, USA). Antibodies against β-TrCP (Cat#4394), Histone H3 (Cat#9715) and ubiquitin (Cat#3933) were from Cell Signaling Technology (Danvers, MA, USA). The protein signals were detected using an ECL chemiluminescence kit (Biological Industries) and the luminescence was visualized using a BioRad luminescent imaging system.

### Nuclear and cytoplasmic protein extraction

The cytoplasmic and nuclear extracts were separated and prepared from cardiac tissue using Nuclear and Cytoplasmic Protein Extraction Kit (Beyotime Biotechnology, China) according to manufacturer's instructions. The hearts were cut into small pieces. Lysis buffer A and B were added and the hearts were grinded into homogenate. The lysates were incubated for 15 min on ice and centrifuged at 1,500 g for 5 min at 4 °C. The supernatant is the cytoplasmic fraction which is confirmed by the expression of GAPDH. The pellets were resuspended in nuclear extraction buffer. After vortexing vigorously on ice, the lysates were centrifuged at 12,000 g for 10 min and the supernatant containing nuclear extracts was collected, and confirmed by the expression of histone H3.

### circHIPK3 deletion by CRISPR/Cas9 approach

C2C12 cells were seeded in plates. After 24 h, the cells were transiently transfected with 4 μg Cas9 plasmid, 4 μg gRNA plasmid using Lipofectamine 2000 (Invitrogen, Carlsbad, CA, USA). The gRNA sequences were listed in Table S2 in the Data Supplement. After 24 h of transfection, the cells were treated with 1 μg/ml puromycin (Merck, USA) and 10 μg/ml blasticidin (Sigma-Aldrich, St Louis, USA) for another 48 hours to select the cells that were successfully transfected.

### Telomere length assay

Genomic DNA was extracted using a tissue and blood DNA extraction kit (Tiangen Biotech, China) according to manufacturer's instructions. Telomere length was determined from total genomic DNA using a qPCR method as previously described 24. The single-copy gene 36B4 was used as a reference. The primers for 36B4 and telomere are shown in Supplemental Table S1. The Ct values for the single-copy gene and telomere length were quantified using SYBR®Premix Ex Taq™. The T (telomere)/S (single-copy gene) ratio was ~[2^Ct(telomere)^/2^Ct(36B4)^]^-1^ (2^-ΔCt^), which reflected the relative length difference in telomeric DNA.

### RNA immunoprecipitation (RIP) assay

The cells were collected and suspended in lysis buffer (Beyotime Biotechnology, China). The entire lysate was incubated with antibodies against HuR (Santa Cruz Biotechnology, Cat#sc-5261), β-TrCP (Cell Signaling Technology, Cat#4394), or IgG (Beyotime Biotechnology, Cat#A7031). RNA-protein complexes were recovered with protein A/G plus-agarose (Santa Cruz Biotechnology, Cat#sc-2003) and then washed with lysis buffer four times. The precipited RNA was analyzed by qRT-PCR.

### Co-immunoprecipitation (Co-IP)

The cells were disrupted by cell lysis buffer for Western blot and IP (Beyotime Biotechnology, China). The lysate was precleared with IgG and protein A/G plus-agarose (Santa Cruz Biotechnology, Cat#sc-2003). Lysates were incubated with the HuR antibody (Santa Cruz Biotechnology, Cat#sc-5261) for 1 hour at 4 °C and then, protein A/G plus-agarose was added and incubated for 1 hour. The expression of HuR, β-TrCP and ubiquitin in whole-cell extracts and immunoprecipitates were analyzed by Western blot.

### RNA-pulldown assay

The RNA-pulldown assay was used to verify the interaction between circHIPK3 and HuR. DNA oligo probes with BiotinTEG at 5' end targeting the back-splice junction of circHIPK3 were designed by RiboBio, and the sequences of circHIPK3 probe were listed in Table S3. After transfection with circHIPK3 plasmid, H9C2 cells were collected and crosslinked with 1% glutaraldehyde at room temperature for 10 min, and then 1.25 M glycine was used to quench the cross-linking reaction. Crosslinked cells were lysed, and sonicated. Chromatin was hybridized with biotinylated probes of circHIPK3 at 37 °C for 4 h. Beads conjugated with streptavidin (Invitrogen, Cat#65001) were added and incubated for 30 min. The eluted protein (HuR) was analyzed by Western blot.

### Construction of lentivirus harboring circHIPK3

The Lentivirus vector plasmid (Geneseed, Guangzhou, China) containing circHIPK3 complementary DNA (cDNA) and packaging plasmids ΔR8.74, VSV-G and Rev. were co-transfected into HEK293T cells as described previously 25.

### Running endurance

Based on our previous protocol 10, before running, mice were acclimated to the treadmill (Jiangsu SANS Biological Technology Co. Ltd.) for 1-2 h and to the motor sound for 15 min. The belt was initially set at a slow speed (6 m/min), then the velocity was increased 2 m every 2 min for the first 12 min and held steady (18 m/min). Exhaustion was defined as the point when mice spent more than 10 consecutive seconds on the shock grid without seeking to re-engage the treadmill.

### Statistical analysis

Data were presented as mean ± SEM. Multiple comparisons were analyzed by ANOVA with Tukey test. Two-tailed t-tests were used to determine the significance of differences between two groups. P < 0.05 was considered statistically significant.

More detailed materials and methods used in this study are provided in the Supplementary material.

## Results

### Profile of circRNAs in young and middle-aged mouse hearts

**Figure 1:**
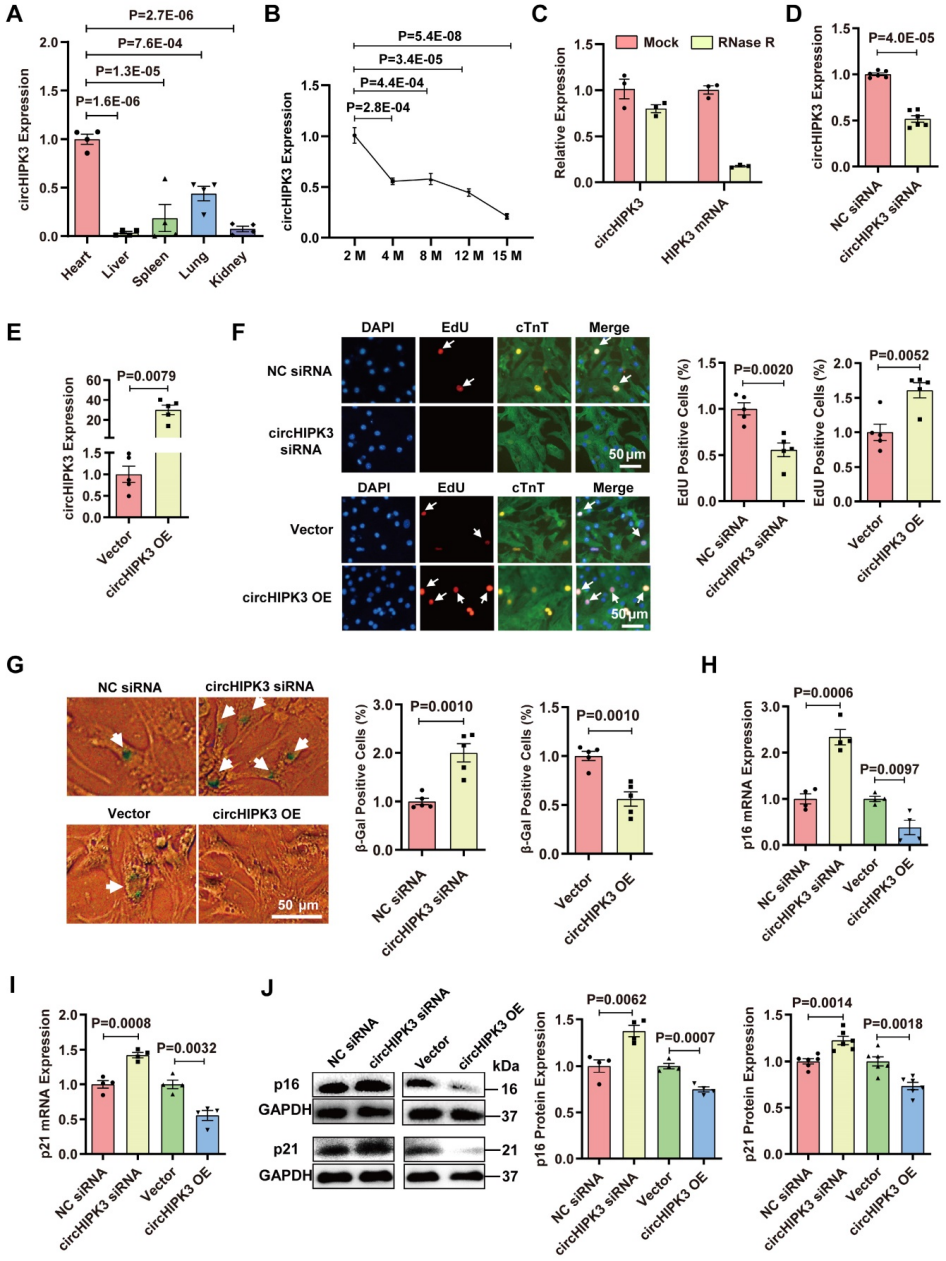
** circHIPK3 inhibits cardiomyocyte senescence. (A)** Quantification of circHIPK3 expression in different mouse tissues. n = 4.** (B)** qRT-PCR analysis of circHIPK3 in the hearts of mice of different ages. n = 4. **(C)** qRT-PCR analysis of the levels of circHIPK3 and HIPK3 in UMSC treated with RNase R. n = 3.** (D)** qRT-PCR analysis of circHIPK3 level in H9C2 cardiomyocyte 40 h after siRNA transfection. n = 6. **(E)** qRT-PCR analysis of circHIPK3 level in H9C2 cardiomyocyte 40 h after transfection of overexpression plasmid. n = 5. **(F)** The proliferation of primary cardiomyocytes was detected by EdU incorporation after transfection with circHIPK3 siRNA or overexpression plasmid for 40 h. n = 5. **(G)** Primary cardiomyocytes transfected with circHIPK3 siRNA or control were subject to β-gal staining, n = 5. **(H-J)** The mRNA and protein levels of p16 and p21 were detected by qRT-PCR or Western blot in H9C2 after transfection with circHIPK3 siRNA or overexpression plasmid for 40 h. n = 4-6. A and B, one-way ANOVA test. D-J two-tailed Student's t test.

We performed RNA sequencing (RNA-seq) using ribosomal RNA-depleted total RNA from young and aging hearts to identify the key molecules and potential signaling pathways triggering cardiac senescence. We found that 95.61% of circRNAs were from protein-coding exons, 2.58% of circRNAs were from intronic, 1.81% of circRNAs were from the unknown region in the young heart. In the middle-aged heart, 95.64% of circRNAs were from protein-coding exons, 2.37% of circRNAs were from intronic, 1.98% of circRNAs were from the unknown region (Figure S1A). Among them, 4028 circRNAs were detected in young mouse hearts, 3834 circRNAs in middle-aged hearts, and 3501 circRNAs were found both in young and middle-aged hearts (Figure S1B). circRNAs were widely distributed uniformly on the chromosomes (Figure S1C). Eighty-eight circRNAs were highly expressed in the young heart, and 98 circRNAs were highly expressed in the middle-aged heart (Figure S1D). circHIPK3, derived from the HIPK3 gene exon 2, is one of the highly expressed circRNAs. circHIPK3 homology analysis in human and mouse genomes was conducted using the Basic Local Alignment Search Tool (BLAST). The results showed the circHIPK3 isoform located at chr11:33307958-33309057 in the human genome (hsa_circ_0000284), and chr2:104310905-104312004 in the mouse genome (mmu_circ_0001052), and circHIPK3 homology is 89% between mouse and human, which is highly conserved. Moreover, the number of circRNAs in middle-aged hearts was smaller than in young hearts (Figure S1E). qRT-PCR assay was used to detect the expression of circHIPK3 in several mouse tissues, including the heart, liver, spleen, lung, and kidney. circHIPK3 expression was found to be most abundantly expressed in the heart (Figure 1A). We then determined the role of circHIPK3 in CM senescence. We found that the expression level of circHIPK3 in the hearts decreased with age (Figure 1B). The data indicate that circHIPK3 may play a significant role in the development of cardiac senescence.

### circHIPK3 inhibits CM senescence

After RNase R exonuclease treatment, HIPK3 mRNA was easily degraded, whereas circHIPK3 was resistant to digestion (Figure 1C and Figure S1F). We found that the siRNA specifically targeting circHIPK3 significantly decreased the circHIPK3 level in H9C2 cells (Figure 1D). We constructed circHIPK3 over-expression plasmid and found that the circHIPK3 level was significantly increased in H9C2 cells after plasmid transfection (Figure 1E). Using gain/loss of circHIPK3 approach, we also demonstrated that the proliferation of primary CMs was decreased by circHIPK3 silencing but increased by its overexpression (Figure 1F). The level of β-Gal staining was increased in the primary CMs when circHIPK3 was silenced but decreased in CMs with circHIPK3 was overexpressed (Figure 1G). The mRNA and protein levels of senescence marker p16 and p21 in H9C2 were significantly decreased by circHIPK3 overexpression, but increased by circHIPK3 silencing (Figure 1H-J). These results suggest that circHIPK3 prevents cell senescence in both H9C2 cell line and primary CMs.

### CM-specific deletion of circHIPK3 in mice induces cardiac senescence

**Figure 2:**
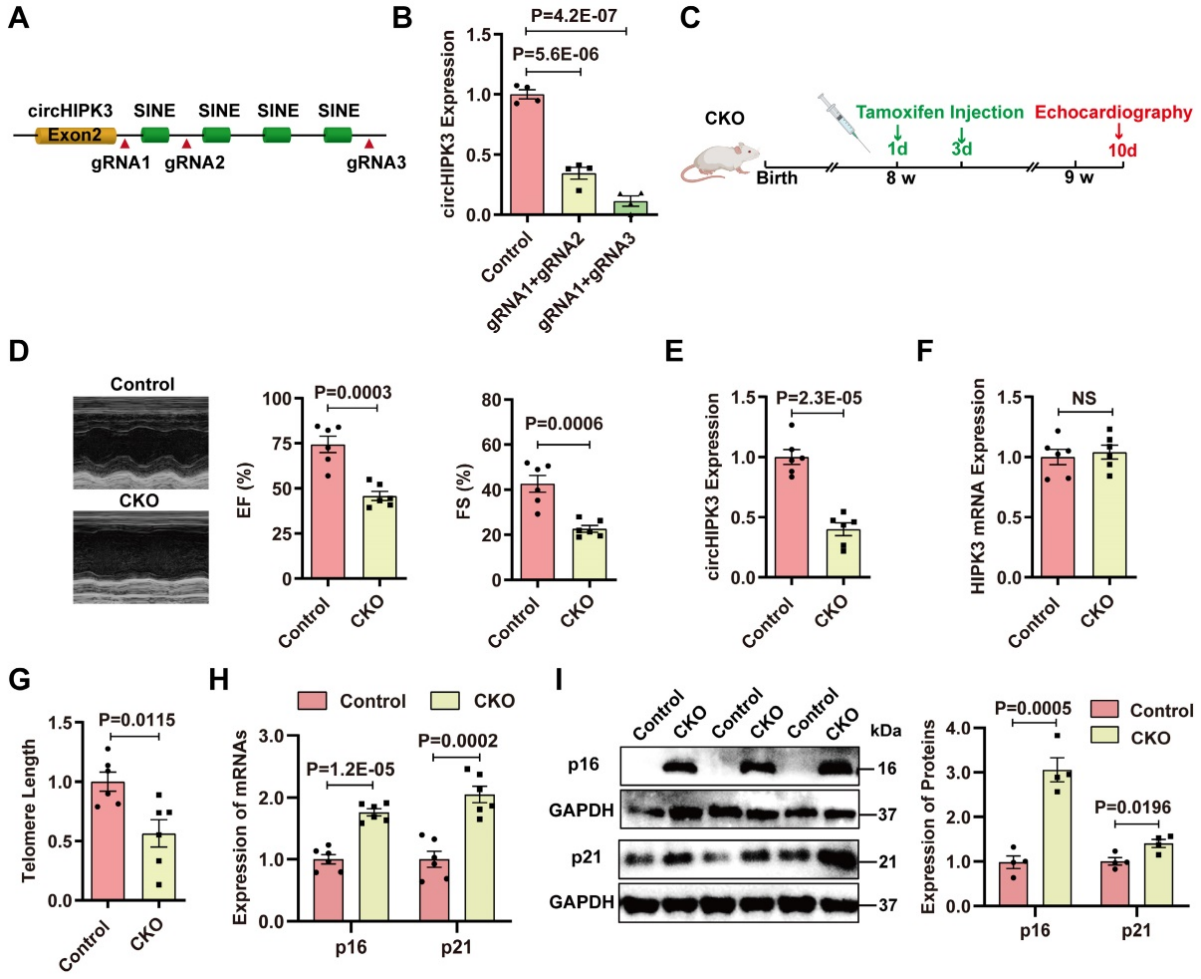
** Specific deletion of circHIPK3 in cardiomyocytes induces cardiac senescence. (A)** SINEs were deleted using CRISPR/Cas9 systems. gRNAs were designed to delete the SINEs sequence.** (B)** qRT-PCR analysis of circHIPK3 expression in cells after SINEs deletion. n = 4. **(C)** Schematic illustration of the procedure to generate cardiomyocyte-specific knockout circHIPK3 mice. 8-week-old mice were subjected to intraperitoneal injection of tamoxifen and the mice were used for subsequent experiment. **(D)** Cardiac function was analyzed by echocardiography in circHIPK3 inducible knockout (CKO) mice after tamoxifen induction and littermate control mice without tamoxifen induction. n = 6. **(E-F)** qRT-PCR analysis of circHIPK3 and HIPK3 mRNA expressions in the hearts of circHIPK3 CKO and control mice. NS, not significant. **(G)** Telomere length was detected using the telomere length assay. n = 6.** (H)** qRT-PCR analysis of p16 and p21 mRNAs expression. n = 6. **(I)** Western blot analysis of p16 and p21 expression in circHIPK3 CKO and control mice. n = 4. B, one-way ANOVA test. D-I, two-tailed Student's t test.

To confirm the role of circHIPK3 in cardiac senescence, we generated CM-specific circHIPK3 knockout (KO) mice. Previous studies have shown that the short interspersed elements (SINEs) of exon 2 are necessary for circHIPK3 circularization 9. CRISPR/Cas9 system was used to delete the downstream SINE sequence of exon 2 in the mouse HIPK3 gene (Figure 2A). The deletion of the downstream SINE sequence of exon 2 in mouse myoblasts significantly inhibited the circularization of circHIPK3 and reduced the level of circHIPK3 as determined by qRT-PCR (Figure 2B). Based on these results, we inserted the loxP sequence into the downstream SINE sequence of exon 2 (Figure S2A). The circHIPK3^Flox/Flox^ mouse was confirmed by Sanger sequencing (Figure S2B-C).

We generated CM-specific circHIPK3 knockout (KO) mice by crossing circHIPK3^Flox/Flox^ mice with αMHC-Cre mice (Figure S2D). The KO mice showed decreased cardiac function (Figure S3A). qRT-PCR confirmed that the expression of circHIPK3 was significantly reduced in the heart of KO mice versus littermate circHIPK3^Flox/Flox^ mice (Figure S3B). Moreover, the KO mice showed telomere length shortening and increased expression of p16 and p21 (Figure S3C-F). These data indicate that circHIPK3 deletion resulted in early cardiac senescence and cardiac dysfunction. However, the reproduction rate of the KO mice was very slow, suggesting that deletion of circHIPK3 impaired development. To overcome this problem, we generated inducible CM-specific circHIPK3 knockout (CKO) mice by crossing αMHC^MerCreMer^ mice with circHIPK3^Flox/Flox^ mice (Figure S4A). The mouse genotype was identified by PCR (Figure S4B-E).

Previous studies reported that Cre enzyme driven by αMHC might impact cardiac cell biology without crossing to a LoxP mouse 26. To determine the potential effect of Cre enzyme, we compared the circHIPK3 expression in the heart and the cardiac function of Cre mice (αMHC^MerCreMer/Wt^ mice with tamoxifen treatment) and CKO mice. The data indicated that CKO mice exhibit decreased circHIPK3 levels in the heart and impaired cardiac function compared to the Cre mice (Figure S4F-G).

Induction of Cre recombinase activity was achieved by tamoxifen administrated intraperitoneally (i.p.) (Figure 2C). Cardiac function of CKO mice was impaired compared to the control mice (littermate without tamoxifen treatment) (Figure 2D). CKO mice exhibited decreased circHIPK3 expression (Figure 2E), while the mRNA level of HIPK3 remains unchanged (Figure 2F). CKO mice displayed telomere length shortening (Figure 2G), which was confirmed in the isolated CMs (Figure S5A-B). Moreover, the mRNA and protein levels of p16 and p21 in the hearts of CKO mice were significantly increased versus control mice (Figure 2H-I). The expressions of hypertrophy marker ANP and BNP were upregulated (Figure S6A-B). The heart weight to body weight ratio was significantly increased (Figure S6C) but the running distance was reduced in the CKO mice (Figure S6D). CKO mice exhibited impaired cardiac function 3 months after tamoxifen induction, and the 3-month survival rate of CKO was decreased to 29.4% (Figure S7A-B). To confirm the protective role of circHIPK3 in cardiac senescence, we injected lentivirus overexpressing circHIPK3 (LV-circHIPK3) in CKO mice (Figure S7C). The LV-circHIPK3 improved CKO cardiac functions (Figure S7D). These results indicate that deletion of circHIPK3 in cardiomyocytes led to cardiac senescence and poor survival.

### circHIPK3 regulates HuR protein expression

**Figure 3:**
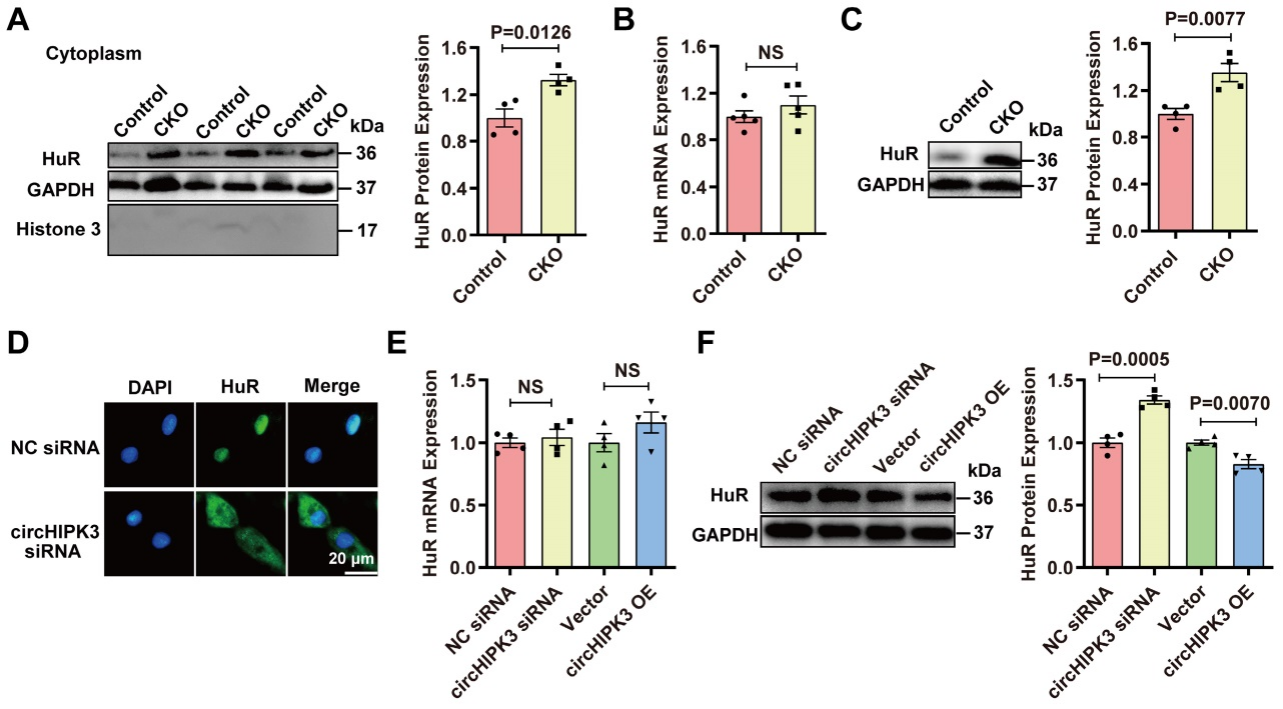
** circHIPK3 regulates HuR protein expression. (A)** Cytoplasm distribution of HuR in control and circHIPK3 knockout mice. n = 4. **(B)** qRT-PCR analysis of HuR mRNA expression in control and CKO mouse heart. n = 5, NS, not significant. **(C)** Western blot analysis of HuR protein level in control and CKO mouse heart. n = 4.** (D)** Localization of HuR by immunofluorescence in H9C2 cells transfected with circHIPK3 siRNA. **(E-F)** qRT-PCR and Western blot analysis of HuR mRNA protein levels in H9C2 cells with circHIPK3 silencing or overexpression. n = 4. Data were analyzed by two-tailed Student's t test.

To understand why circHIPK3 deletion resulted in upregulation of p21, we analyzed the possible target protein of circHIPK3 through the circular RNA interaction website (*https://circinteractome.nia.nih.gov/*). The analysis did not find p21 or p16 as the target for circHIPK3 but showed that there were three binding sites between HuR and circHIPK3 in humans (Figure S8A). RPISeq analysis (*http://pridb.gdcb.iastate.edu/RPISeq/*) confirmed that circHIPK3 binds to HuR in mice (Figure S8B), and HuR protein was highly conserved between humans and mice (Figure S8C). The analysis through the protein-protein interaction (PPI) database (*https://www.string-db.org/*) revealed that HuR is involved in the development of senescence by modulating SIRT1, cell cycle-related proteins, and telomere length (Figure S8D-E), which suggests that HuR protein may be directly involved in the aging process. Importantly, HuR binds to the AU-rich element (ARE) in the 3'-UTR region of p21 mRNA to increase its stability and eventually lead to the enhanced expression of p21 protein in the cytoplasm 27, 28. Previous studies have shown that HuR is mainly located in the nucleus, but it can be translocated into the cytoplasm under ischemia, hypoxia, and ultraviolet irradiation 28, 29. Therefore, we hypothesized that circHIPH3 prevents senescence by either inhibiting the translocation of HuR to the cytoplasm or increasing its degradation, leading to the destabilization of p21 mRNA. To exam this hypothesis, we performed loss and gain of function experiments. HuR was increased in cytoplasm fractions from hearts of CKO mice as determined by Western blot (Figure 3A). Interestingly, the expression of HuR mRNA in the hearts between control and circHIPK3 CKO mice was not different, but the level of HuR protein was upregulated in CKO mouse hearts (Figure 3B-C). HuR was increased significantly in the cytoplasm of H9C2 cells transfected with circHIPK3 siRNA as determined by immunofluorescence (Figure 3D). The expression of HuR mRNA in H9C2 cells was unaltered by either silencing or overexpressing circHIPK3 (Figure 3E). The total protein level of HuR was decreased in H9C2 cells when circHIPK3 was overexpressed, but it was increased when circHIPK3 was silenced (Figure 3F). Together, these results suggest that circHIPK3 can determine the localization of HuR, and regulate the expression of HuR at the post-transcription level.

### circHIPK3 acts as a scaffold for HuR and E3 ubiquitin ligase and promotes its degradation

**Figure 4:**
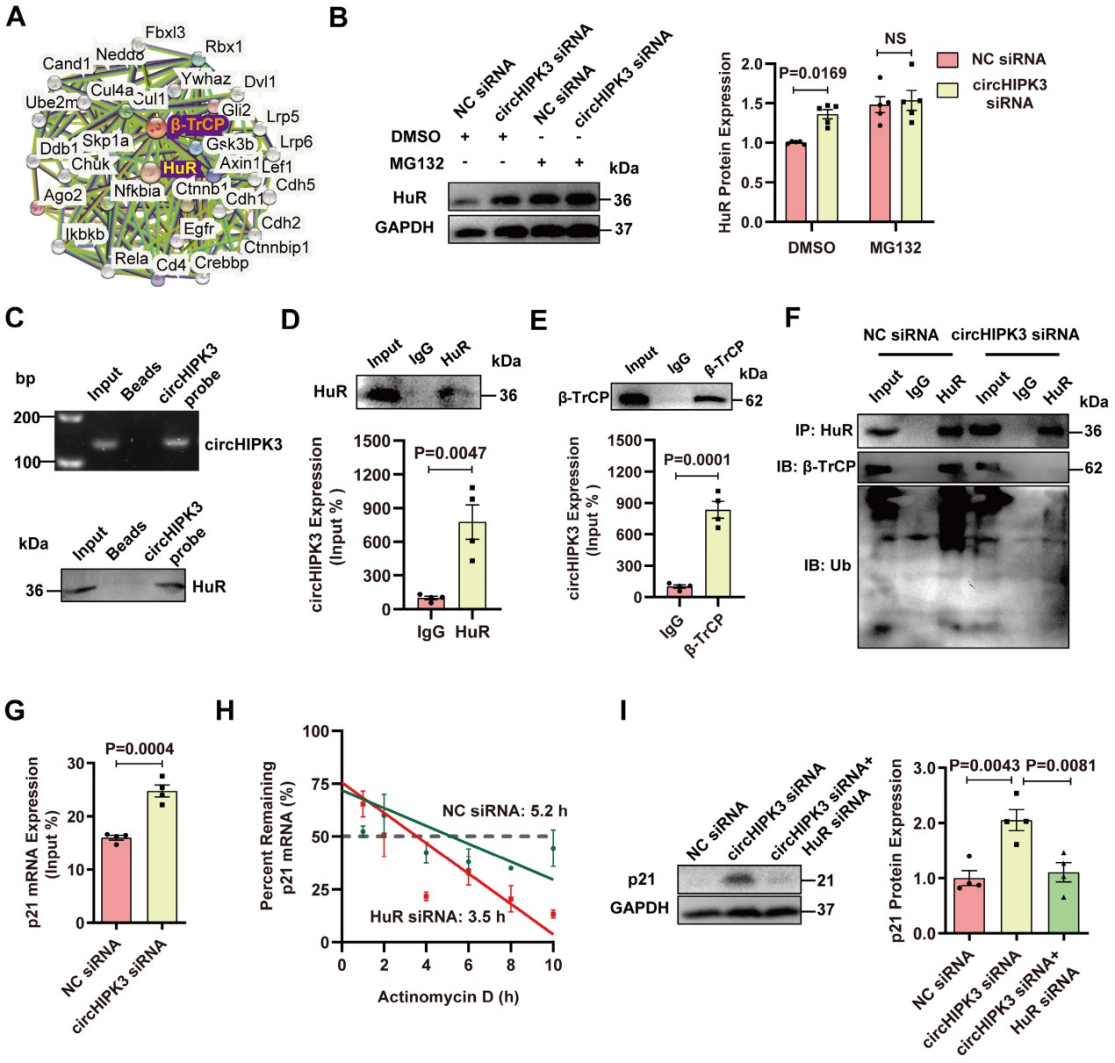
** circHIPK3 promotes HuR degradation by recruiting E3 ubiquitin ligase. (A)** The interaction network of HuR and β-TrCP protein was analyzed by the STRING database. **(B)** Western blot analysis of HuR in H9C2 cells transfected with circHIPK3 siRNA cells with or without MG132 treatment for 6 h. n = 5.** (C-D)** H9C2 cells were transfected with circHIPK3 overexpression plasmid for 40 h. RNA-pull down assay **(C)** and RIP **(D)** experiments were performed to demonstrate the interaction between circHIPK3 and HuR. n = 4.** (E)** RIP experiment showed the interaction between circHIPK3 and the ubiquitin E3 ligase β-TrCP. n = 4.** (F)** The cells were transfected with circHIPK3 siRNA for 40 h. Co-IP experiment showed that the interaction between HuR and β-TrCP was attenuated and the binding of HuR and ubiquitin was decreased by circHIPK3 siRNA. n = 4.** (G)** H9C2 cells were transfected with circHIPK3 siRNA for 40 h. RIP experiment showed that the interaction between HuR and p21 mRNA was enhanced by circHIPK3 silence. n = 4.** (H)** The half-life of p21 mRNA in NC siRNA and HuR siRNA groups was assessed 40 h after transfection by treating the cells with actinomycin D (2 mg/ml); mRNA half-life was calculated by qRT-PCR. n = 3. **(I)** Western blot analysis of p21 protein in H9C2 transfected with NC siRNA, circHIPK3 siRNA and circHIPK3 siRNA+HuR siRNA. n = 4. B, two-way ANOVA test. D, E, G, two-tailed Student's t test. I, one-way ANOVA test.

Previous studies showed that HuR could be degraded by the ubiquitin-proteasome pathway through the interaction with E3 ubiquitin ligase β-TrCP 14, 15. Indeed, an interaction between HuR and E3 ubiquitin ligase β-TrCP can be found in the PPI database (Figure 4A). We sought to determine whether circHIPK3-induced downregulation of HuR protein is mediated by the ubiquitin-proteasome system. H9C2 cells were treated with a specific proteasome inhibitor MG132 to block the degradation of proteins through the proteasome. MG132 treatment enhanced the accumulation of HuR in the cytoplasm induced by circHIPK3 knockdown (Figure 4B), suggesting that HuR is degraded by the proteasome, and the relative higher level of HuR in circHIPK3 knockdown cells is due to the reduction of HuR degradation. The physical association between circHIPK3 and HuR by RNA-pull down-Western blot assay (Figure 4C). RIP experiments and qRT-PCR revealed that circHIPK3 was present in the HuR pulldown sample (Figure 4D), suggesting that circHIPK3 may regulate HuR stability through direct interaction. Therefore, we further investigated whether circHIPK3 regulated HuR degradation via the ubiquitin-proteasome system. RPISeq prediction revealed that circHIPK3 not only can bind to HuR, but also can bind to ubiquitin ligase β-TrCP (Figure S8F). RIP assay confirmed that there was a direct interaction between circHIPK3 and β-TrCP (Figure 4E). Co-IP showed that circHIPK3 silencing weakened the interaction between HuR and β-TrCP, and reduced HuR ubiquitination (Figure 4F). RIP experiment revealed that circHIPK3 silencing enhanced the interaction between HuR protein and p21 mRNA (Figure 4G). To determine the role of HuR in p21 mRNA expression, we treated the cells with an RNA synthesis inhibitor (actinomycin D), and found that the half-life p21 mRNA declined from 5.2 h to 3.5 h after HuR silencing (Figure 4H), indicating that HuR can increase p21 mRNA stability. To determine whether circHIPK3 silencing promotes p21 expression by inhibiting HuR, we knocked down circHIPK3 and HuR with siRNA. Western blot showed that HuR silencing reversed the effect of circHIPK3 siRNA on p21 expression, indicating that circHIPK3 inhibits p21 level by regulating HuR (Figure 4I). These results demonstrate that circHIPK3 enhances HuR and β-TrCP interaction, resulting in the degradation of HuR through the ubiquitin-proteasome pathway, leading to reduced expression of p21.

### Exosomes prevent CM senescence by releasing circHIPK3

**Figure 5:**
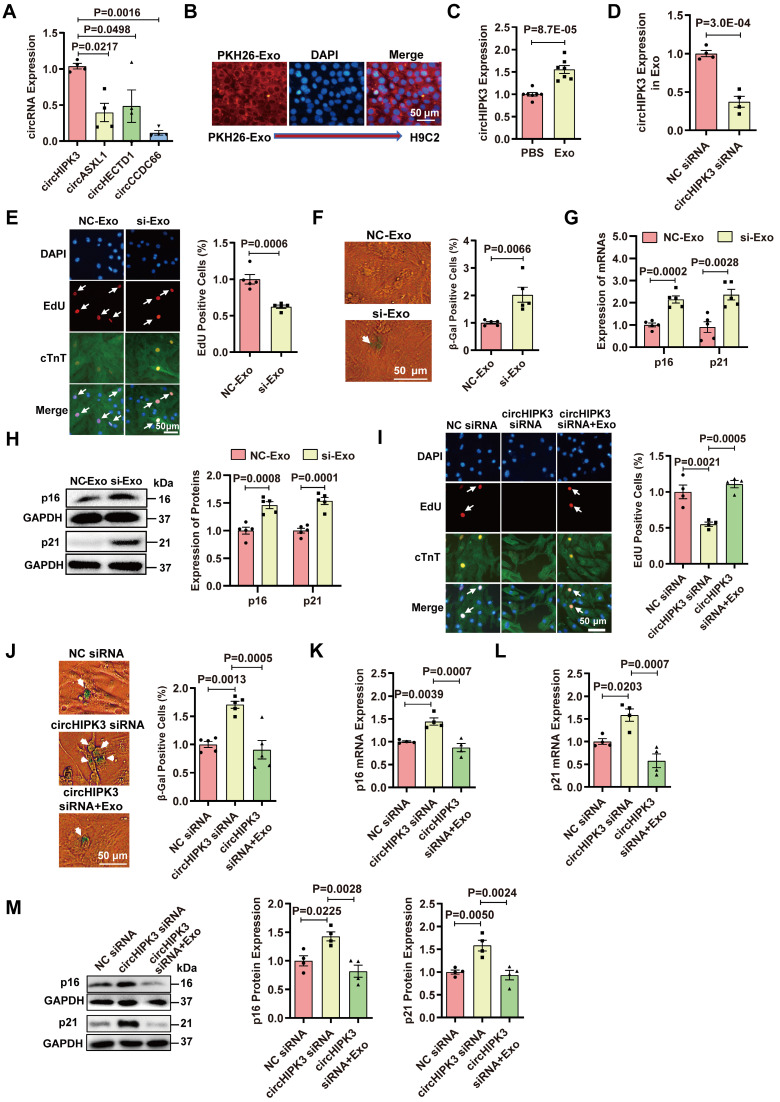
** Exosomes prevent cardiomyocyte senescence by releasing circHIPK3. (A)** The expression of circRNAs in exosome was determined by qRT-PCR analysis. n = 4. **(B)** PKH26-labeled Exo uptake was detected by fluorescence microscopy. n = 6.** (C)** qRT-PCR analysis of the expression of circHIPK3 in H9C2 cells treated with exosome for 24 h. n = 7. **(D)** circHIPK3 siRNA transfection in UMSC resulted in decreased expression of circHIPK3 in exosome. n = 4. **(E)** The proliferative rate of primary cardiomyocytes treated with NC-Exo or si-Exo (exosomes isolated from UMSC transfected with circHIPK3 siRNA). n = 5. **(F)** β-gal staining for primary cardiomyocytes treated with NC-Exo or si-Exo. n = 5. **(G-H)** qRT-PCR and Western blot analysis of p16, p21 mRNAs and proteins in H9C2 cells treated with NC-Exo or si-Exo for 24 h. n = 5. **(I)** EdU staining of proliferating primary cardiomyocytes treated with NC siRNA, circHIPK3 siRNA, and circHIPK3 siRNA+Exo. n = 4.** (J)** β-gal staining for primary cardiomyocytes treated with NC siRNA, circHIPK3 siRNA and circHIPK3 siRNA+Exo. n = 5. **(K-M)** The mRNA and protein levels of p16 and p21 were detected by qRT-PCR and Western blot in H9C2 cells treated with NC siRNA, circHIPK3 siRNA, and circHIPK3 siRNA+Exo group. n = 4. A, one-way ANOVA test. C-H, two-tailed Student's t test. I-M, one-way ANOVA test.

Exosomes contain noncoding RNAs including microRNA, lncRNA, and circRNA 19, 20. Our previous studies have demonstrated that UMSC-Exos inhibit aging-induced vascular dysfunction and cardiac dysfunction 21, 22. We sought to determine whether the beneficial effect could be mediated by circHIPK3. The characteristics of exosomes were confirmed by measuring the expression of exosome-specific markers CD63 and CD9 via flow cytometry and Western blot analysis (Figure S9A-B). The particle sizes and numbers were measured by NanoSight analysis, which showed that exosomes were approximately 30-100 nm in diameter (Figure S9C). We screened several proliferation-related circRNAs, including circHIPK3 30, circASXL1 31, circHETD1 32, circCCDC66 33, and found that circHIPK3 was enriched in exosome (Figure 5A). Exosomes were labeled with PKH26 and co-incubated with H9C2 for 24 hours. PKH26-labeled Exo uptake was detected by fluorescence microscopy (Figure 5B). Exosomes co-incubation resulted in the upregulation of circHIPK3 expression in H9C2 cells (Figure 5C). To determine whether exosomes exert a protective effect by releasing circHIPK3, we silenced circHIPK3 in exosomes (Figure 5D). The results showed that exosomes promote H9C2 cell proliferation, inhibit senescence and decrease the expressions of p16 and p21. However, the protective effect of exosomes against senescence was abolished by circHIPK3 silencing (Figure 5E-H). These results suggest that exosomes promote H9C2 cell proliferation and inhibit their senescence by delivering circHIPK3. To further confirm these findings, we silenced circHIPK3 in primary CMs with siRNA, and then treated the CMs with exosomes for 24 h. EdU experiment showed that circHIPK3 silencing inhibited the proliferation of primary CMs (Figure 5I), and promoted senescence (Figure 5J) which was reversed by exosomes. Furthermore, the expression of p16 and p21 was increased by silencing circHIPK3, and the effect was alleviated by exosomes (Figure 5K-M).

### Exosomes prevent cardiac senescence in circHIPK3 knockout mice

**Figure 6:**
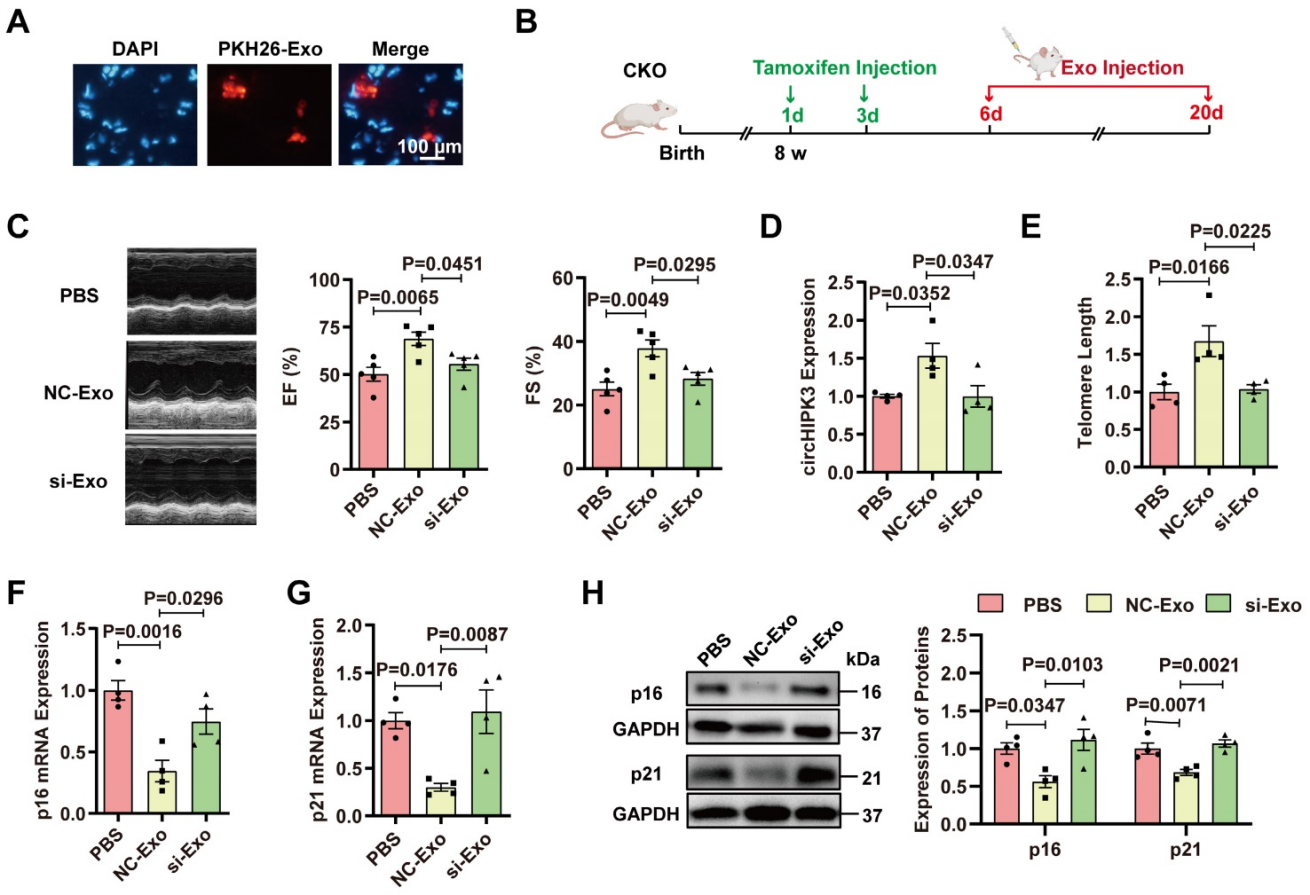
** Exosomes prevent cardiac senescence in circHIPK3 knockout mice. (A)** PKH26-labeled exosomes were injected via the tail vein. After 30 min, the hearts were harvested and the labeled exosomes in hearts were observed by fluorescence microscopy. **(B)** Schematic illustration of experiments performed in panels C-H. 8-week-old mice were subjected to intraperitoneal injection of tamoxifen and the mice were injected with exosome via the tail vein into CKO mice three times a week. By day 20, the mice were subjected to subsequent experiment. **(C)** Cardiac function analyzed by echocardiography in circHIPK3 CKO mice injected with PBS, NC-Exo and si-Exo. n = 5. **(D)** qRT-PCR analysis of circHIPK3 in circHIPK3 CKO mice injected with PBS, NC-Exo and si-Exo. n = 4. **(E-G)** qRT-PCR analysis of telomere length assay, p16 and p21 mRNAs in circHIPK3 CKO mice injected with PBS, NC-Exo and si-Exo. n = 4. **(H)** Western blot of p16 and p21 proteins in circHIPK3 CKO mice injected with PBS, NC-Exo and si-Exo. n = 4. Data were analyzed by one-way ANOVA test.

To determine whether cardiac senescence in the circHIPK3 CKO mice can be prevented by exosomes, PKH26-labeled exosomes were injected via the tail vein. The fluorescence of PKH-26 in the heart was observed under the microscope 30 minutes after injection (Figure 6A). To determine whether exosomes protect the heart against cardiac senescence by delivering circHIPK3, we injected PBS, exosome (NC-Exo), circHIPK3 silenced exosome (si-Exo) via the tail vein (100 μg) into CKO mice three times a week. After two weeks, the effect of exosomes was evaluated (Figure 6B). Mice injected with exosomes exhibited improved cardiac function (Figure 6C), increased circHIPK3 level (Figure 6D), longer telomere length (Figure 6E), and decreased mRNA and protein levels of p16 and p21 (Figure 6F-H). However, these beneficial effects exerted by exosomes were inhibited by silencing circHIPK3 (Figure 6C-H). Moreover, the exosome administration also attenuated the deterioration of the heart function in KO mice (Figure S10).

## Discussion

**Figure 7:**
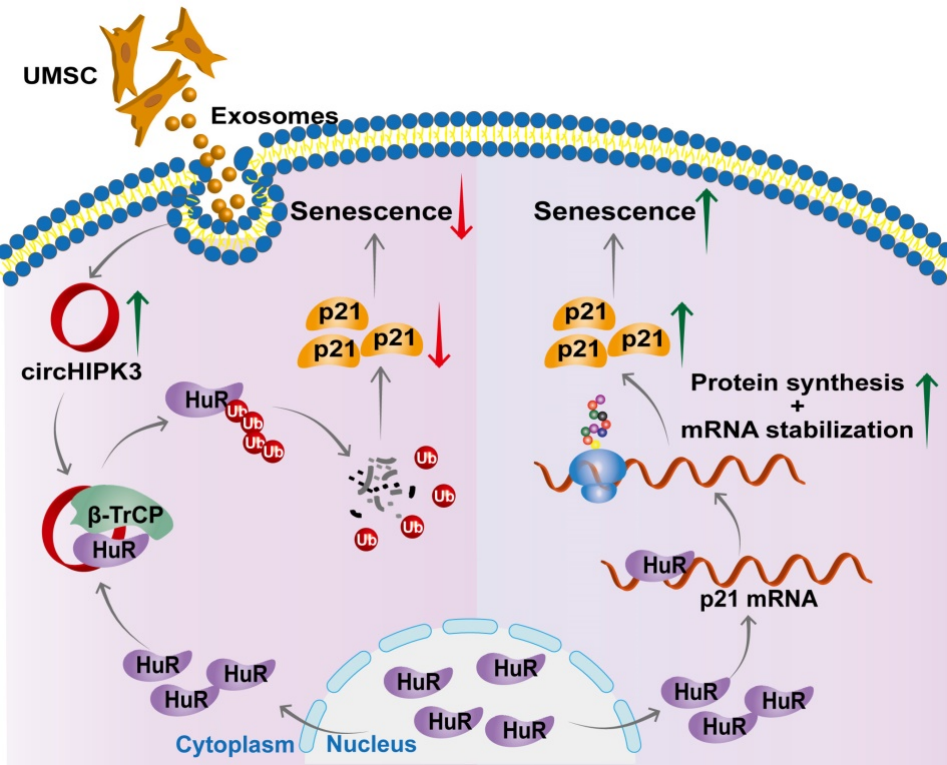
** Proposed mechanisms of circHIPK3/HuR/p21 regulation in cardiac premature senescence.** HuR in the cytoplasm binds to AU-rich elements in the 3'UTR of p21 mRNA, promoting the expression of p21 protein. Exosomal circHIPK3 binds to HuR and β-TrCP to promote the degradation of HuR via the ubiquitin-proteasome pathway. Increased circHIPK3 protects heart from premature senescence through destabilizing HuR protein and decreasing p21 expression.

In this study, we successfully generated inducible CM-specific circHIPK3 knockout (CKO) mice and showed that CKO mice exhibited increased CM senescence and decreased cardiac function. We also found that circHIPK3 is directly bound to HuR and E3 ligase β-TrCP, acts as scaffold and promotes the degradation of HuR *via* the UPS. circHIPK3 thereby prevents the binding between HuR and p21, reduces the stability of p21 mRNA, and inhibits cardiac senescence (Figure 7). Importantly, we demonstrated that UMSC-Exos improved the cardiac function of CKO mice by delivering circHIPK3.

A suitable mouse model is essential for aging research. However, current models have several disadvantages. The spontaneous aging model needs a long time to develop the phenotype, and the D-gal administration-induced model requires long-term administration of the chemical 34. Therefore, our CKO model provides an effective and convenient tool for aging research. In this study, we found that the expression of circHIPK3 was decreased in the aging heart. A decreased circHIPK3 level is associated with cellular senescence. CircHIPK3 consists of the second exon from the HIPK3 gene flanked by short interspersed elements (SINEs) on either side to secure its circularization 9. We showed that the circHIPK3 level is significantly reduced in mouse myoblasts when the downstream SINE sequence is deleted. Therefore, the SINE sequence is required for the formation of circHIPK3. Based on these studies, we knocked out the downstream SINE sequence to generate CM-specific circHIPK3 knockout (KO) mice, which showed increased p16 and p21 expression, telomere shortening, and decreased cardiac function. However, the reproductive rate of these mice was very slow, suggesting that circHIPK3 is important for cardiac development.

Because we were not able to generate a sufficient number of KO mice for the study, we generated inducible CM-specific circHIPK3 knockout (CKO) mice for subsequent experiments. Similar to the circHIPK3 KO mice, the CKO mice also showed exaggerated CM senescence and impaired cardiac function. Thus, our circHIPK3 KO mice provide a new potential model for congenital heart disease, and inducible circHIPK3 CKO mice are a promising model for senescence-induced cardiac dysfunction. To our knowledge, our CM-specific KO mice and inducible circHIPK3 CKO mice are novel circRNA models for research on stage-specific cardiac development.

Moreover, one report showed that dystrophin and calcium current decreased in cardiomyocytes expressing Cre enzyme driven by αMHC but not TNT promoter (rat troponin T2 cardiac promoter) 26. The differences in phenotype between αMHC-Cre and TNT-Cre mice may be due to genetic background and the “time window” of Cre activity. In response to Cre expression, mice on mixed backgrounds are more resistant to developing cardiac dysfunction than mice on a pure background 35. TNT-Cre is mainly expressed during embryogenesis, and the Cre was expressed only in newborn TNT-Cre mouse hearts. Therefore, TNT-Cre mice are useful and have been used to generate early cardiomyocyte-specific mutants. Moreover, previous studies showed that some cardiac stem or progenitor cells, such as c-Kit^+^ cells or BCRP^+^ cells, may express TNT 36, 37. It is possible that TNT-Cre may knockout target genes in stem cells or progenitors. In our study, circHIPK3 was knocked out in adult mice. Similar to other groups, we chose a more specific and mature Cre line driven by αMHC, which will not contaminate any noncardiomyocyte lineages 38-40. A previous study reported that before six months of age, αMHC-Cre and WT mice had identical cardiac structure and function, which indicated that the impacts of αMHC-Cre on cardiac cell biology depend on age 40. Our study focused on 2-month-old mice, and the results showed that αMHC-Cre did not impact cardiac cell biology.

It was also reported that there were no alterations in cardiac function in αMHC^MerCreMer^ transgene either with or without tamoxifen treatment 41. Other studies indicated that αMHC^MerCreMer^ might cause cardiotoxicity depending on the dose and the frequency of tamoxifen injected, but tamoxifen alone had no effect on cardiac structure or function, regardless of the dose used 42, 43. They 43 injected tamoxifen intraperitoneally on three consecutive days at doses 90 μg/g body weight/day in 6-week-old mice. We injected tamoxifen in 8-week-old mice twice, but not on consecutive days based on the study by Huang et al d 44. We also used αMHC-Cre mice as control and found that CKO mice showed reduced circHIPK3 levels in the hearts and worsening cardiac function, but αMHC-Cre mice had normal heart function. The data were consistent with other groups' reports 42, 43.

Of note, circHIPK3 is a double-edged sword in cardiovascular disease. Recent studies reported that circHIPK3 promotes angiogenesis and induces cardiomyocyte proliferation to improve cardiac function after myocardial infarction in mice 7, 8, which align with our findings. However, one study showed that silencing circHIPK3 in cardiac fibroblasts inhibited cardiac fibrosis and improved cardiac function in diabetes cardiomyopathy 45. Another paper suggests that circHIPK3 serves as a miR-29b-3p sponge to stimulate cardiac fibroblasts proliferation, migration, and development of cardiac fibrosis in angiotensin II-induced heart injury 46. These studies indicate that circHIPK3 may have a different function in different cell types. It would be interesting to generate fibroblast-specific circHIPK3 knockout mice to explore the effect of circHIPK3 in cardiac fibrosis.

Based on differences in the composition and origin of sequences, circRNAs can be divided into three categories: exonic (ecircRNA), exonic-intronic (eicircRNA), and intronic (ciRNA). EicircRNAs and ecircRNA are preferentially located in the cytoplasm, but ciRNAs are predominantly located in the nucleus 9, 47. CircHIPK3 is solely generated from the second exon and therefore belongs to the ecircRNA category. In this study, we demonstrated that circHIPK3 enhanced the interaction between E3 ubiquitin ligase β-TrCP and HuR protein in the cytoplasm, which is consistent with previous studies showing that circHIPK3 is preferentially localized in the cytoplasm 9, 30. It is known that circRNAs play a critical role in cardiac development and diseases, including myocardial infarction, pathological hypertrophy, and heart failure 48-50. Most studies have shown that circRNAs act as a microRNA sponge, our finding that circHIPK3 acts as a scaffold for HuR and E3 ubiquitin ligase β-TrCP, and promotes the ubiquitination and degradation of HuR in the cytoplasm is novel and sheds new light on the circRNA-mediated molecular mechanisms underlying cardiac senescence.

Previous studies have shown that HuR is primarily localized in the nucleus, where it plays a protective role in certain types of cells. It was reported that HuR could directly bind to the telomerase RNA component (TERC), promote the assembly of the TERC/hTERT complex, improve telomerase activity, and ultimately enhance the growth of hematopoietic stem cells 51. Moreover, Wang *et al*. found that knocking out HuR in the heart can aggravate isoproterenol-induced cardiac remodeling 52. Therefore, HuR is essential for maintaining the normal function of cells. However, HuR was found to accumulate in the heart 3 days after myocardial infarction, exacerbate inflammation and decrease cardiac function in mice 53, 54. Additionally, one study showed that HuR relocates to the cytoplasm from the nucleus to disrupt the expression of CUGBP1. Restoration of CUGBP1 expression protects cardiomyocytes from ischemia-induced injury via promoting angiogenesis and decreasing apoptosis through activating the VEGF-A gene 11. Therefore, the relocation of HuR in the cytoplasm is responsible for impaired cardiac function through the regulation of CUGBP1 expression 11. In our study, HuR translocates into the cytoplasm and binds to AU-rich elements in the 3'UTR of p21 mRNA, enhancing the aging-inducer p21 stability and function. Our study indicates that HuR located in the cytoplasm impairs cell function. Therefore, we propose that HuR in the nucleus is essential to maintain physiological function, but its accumulation in the cytoplasm may impair cell functions. Together, the studies support the hypothesis that the location of a molecule determines its function.

Furthermore, we showed that circHIPK3-mediated HuR degradation in the cytoplasm leads to decreased p21 mRNA stability. p21 is the cyclin-dependent kinase inhibitor 55, and upregulation of p21 induces senescence in fibroblasts 56 and dopaminergic neurons 57. Our findings support p21 as an inducer of senescence and show that HuR acts as a stabilizer of p21 mRNA, and therefore identified a new mechanism explaining how p21 is down-regulated by circHIPK3 in the cytoplasm. We, for the first time, showed that circHIPK3 acts as a scaffold to bring HuR to E3 ligase β-TrCP together and promote its ubiquitination and degradation, leading to decreased p21 mRNA stability in the cytoplasm. We found a strong interaction between HuR protein and p21 mRNA. Moreover, Sturmlechner et al. showed that one-third of the aging-related gene expression is p21 dependent 58. Furthermore, it was shown that p16 expression is regulated by p21 59-61. Thus, we focused on how to explore how circHIPK3 regulates p21 expression in this study.

Aging is a significant risk factor for the development of cardiac disease, yet no therapeutic approach is currently available. Chemical inhibitors have been used to disrupt HuR binding to the TNF-α mRNA 62, 63. However, these inhibitors lack specificity. Our findings that circHIPK3 acts as scaffold between E3 ubiquitin ligase and the target protein HuR provide a new strategy to inactivate senescence-inducer p21. Importantly, several studies have demonstrated that exosomes have a protective effect on myocardial infarction, ischemia/reperfusion injury, and heart failure 64-66. Moreover, the changes of circulating circHIPK3 in the blood exosome with age have the potential to determine aging-related cardiac dysfunction. Due to their unique features, such as low immunogenicity and high stability, exosomes can be used as natural vehicles for the delivery of therapeutic ncRNAs. Therefore, exosome-based therapy is emerging as a promising strategy for restoring cardiac function after injury. In this study, we showed that UMSC-Exos prevent CM senescence and improve cardiac function by releasing circHIPK3, which has clinical implications in treating aging-induced cardiac dysfunction.

In summary, we successfully generated CM-specific circHIPK3 knockout mice and demonstrated that deletion of circHIPK3 led to decreased cardiac function and exaggerated CM senescence. As a scaffold, circHIPK3 enhanced the binding of E3 ubiquitin ligase β-TrCP and HuR in the cytoplasm, leading to its ubiquitination and degradation. Reduced HuR in the cytoplasm resulted in decreased stability of senescence-inducer p21 mRNA and its activity. Importantly, the anti-senescence and cardio-protective effect exerted by UMSC-Exos is mediated by circHIPK3. These findings pave the way for the development of new therapies for aging-associated cardiac dysfunction.

## Supplementary Material

Supplementary methods, figures and tables.

## Abbreviations

ARE: AU-rich element
CM: cardiomyocyte
circRNA: circular RNA
CKO: CM-specific tamoxifen-induced circHIPK3 knockout
Exo: exosome
HuR: human antigen R
SINE: short interspersed element
UMSC: umbilical cord mesenchymal stem cell
UPS: ubiquitin-proteasome system
β-TrCP: β-transducin repeat-containing protein
