# Supplementary material for: circHIPK3 prevents cardiac senescence by acting as a scaffold to recruit ubiquitin ligase to degrade HuR
Source: Theranostics. 2022 Oct 31;12(17):7550–66. doi: 10.7150/thno.77630 (PMC9691369; doi:10.7150/thno.77630)
Supplement: Supplementary file 1 — Supplementary methods, figures and tables. [file thnov12p7550s1.pdf]

circHIPK3 prevents cardiac senescence by acting as a scaffold to recruit ubiquitin ligase to degrade HuR

Fengzhi Ding[1]*, Lin Lu[1]*, Chengjie Wu[1]*, Xiangbin Pan[2]*, Bin Liu[3], Yu Zhang[1], Yanli Wang[1], Weiliang Wu[1], Bing Yan[1], Yuqing Zhang[1], Xi-Yong Yu[4], Yangxin Li[1#]

[1]Institute for Cardiovascular Science and Department of Cardiovascular Surgery, First Affiliated Hospital and Medical College of Soochow University, Collaborative Innovation Center of Hematology, Soochow University, Suzhou, Jiangsu 215123, P. R. China

[2]Department of Structural Heart Disease, National Center for Cardiovascular Disease, China & Fuwai Hospital, Chinese Academy of Medical Sciences & Peking Union Medical College, Beijing, Key Laboratory of Cardiovascular Appratus Innovation, Beijing 100037, P.R. China

[3]Department of Cardiology, the Second Hospital of Jilin University, Changchun, Jilin 130041, P. R. China

[4]Key Laboratory of Molecular Target & Clinical Pharmacology and the NMPA ＆ State Key Laboratory of Respiratory Disease, Guangzhou Medical University, Guangzhou, Guangdong 511436, P. R. China

Corresponding author: Yangxin Li, PhD, FAHA, Institute for Cardiovascular Science and Department of Cardiovascular Surgery, First Affiliated Hospital of Soochow University, Suzhou, Jiangsu 215123, P. R. China. Tel: 86-512-67781962, or Fax: 86-512-67780100, Email: yangxin_li@yahoo.com (YL).

*These authors contributed equally to this work.

[#]Corresponding author.

Figure S1

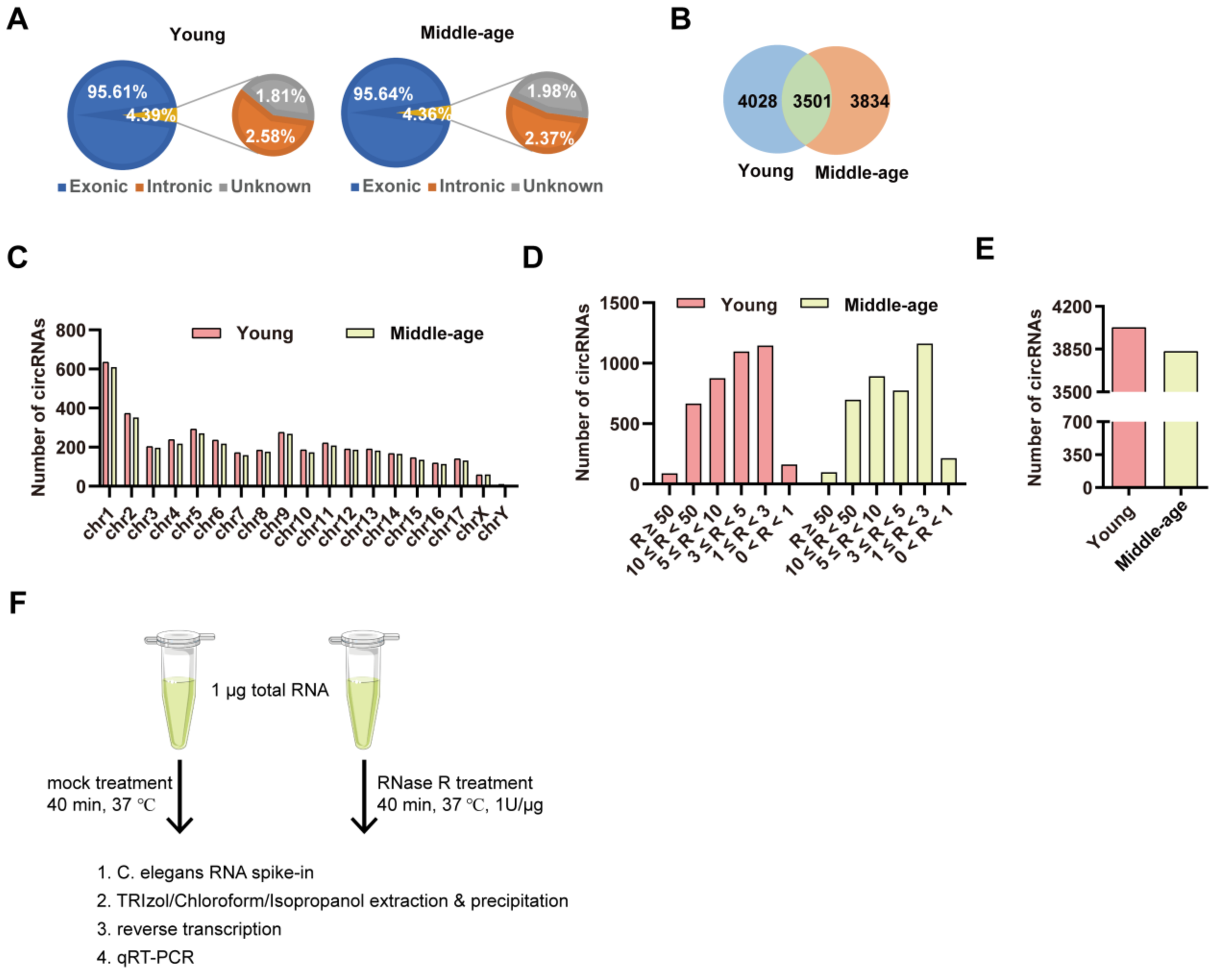

Figure S1 Profile of circRNA expression in young and middle-aged mouse hearts. (A) RNA-seq analyses of circRNA from young and middle-aged hearts. Reads distribution of circRNA in genome. The circRNAs identified from exonic, intronic, and unknown are shown in “Blue”, “Orange” and “Brown”, respectively. **(B)** Venn diagram of circRNAs expression in young and middle-aged groups. **(C)** The quantity of circRNAs derived from different chromosomes. The circRNAs identified in young and middle-aged samples are shown in “pink” and “yellow”, respectively. **(D)** Read number of circRNA-seq analyses. **(E)** The number of circRNAs in young and middle-aged hearts detected by circRNA-seq. **(F)** The flow chart of RNase R treatment performed in Figure 1C.

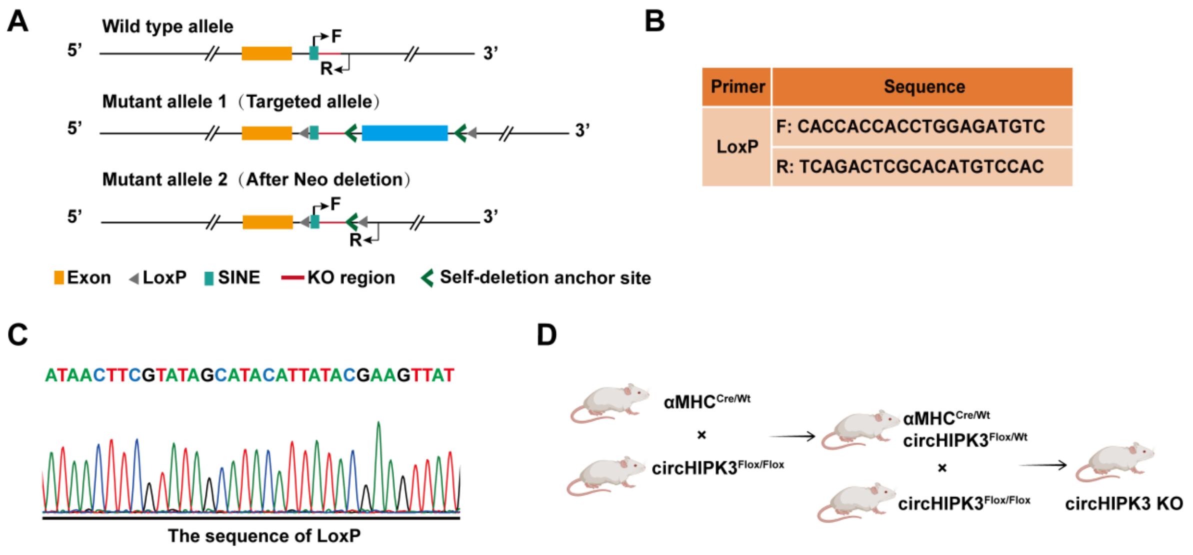

Figure S2 Generation of cardiomyocyte-specific circHIPK3 knockout mice. (A) Construction strategy for circHIPK3 knockout (KO) mice. **(B-C)** Genetic identification of Flox mice by DNA sequencing. **(D)** Schematic illustration of the breeding strategy to generate KO mice.

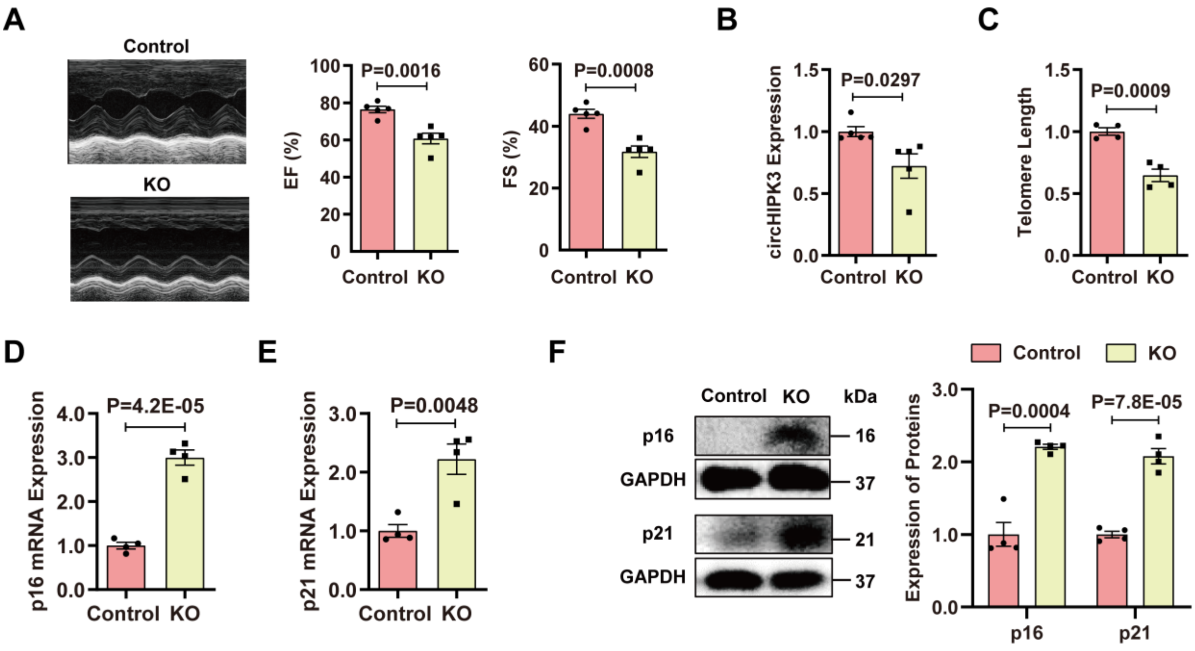

Figure S3 Deletion of circHIPK3 inhibits cardiac function. (A) Cardiac function analyzed by echocardiography for 8-week-old circHIPK3 knockout (KO) mice. n = 5. **(B)** qRT-PCR analysis of circHIPK3 expression. n = 5. **(C)** Telomere length of the hearts from control and KO mice was determined by telomere length assay. n = 4. **(D-E)** qRT-PCR analysis of cardiac p16 and p21 mRNAs in control and circHIPK3 KO mice. n = 4. **(F)** Western blot analysis of cardiac p16 and p21 proteins in control and circHIPK3 KO mice. n = 4. Data were analyzed by two-tailed Student’s t test.

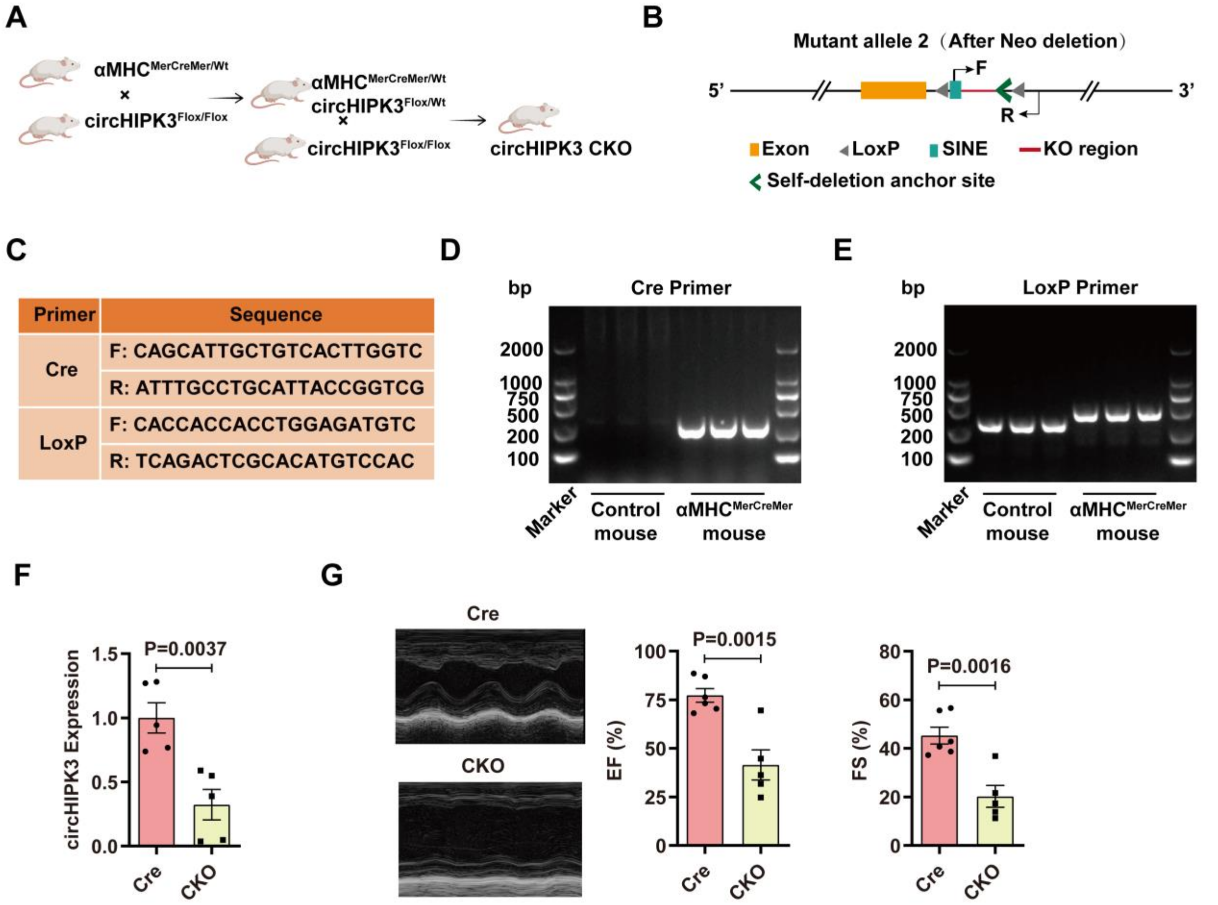

Figure S4 Generation of inducible cardiomyocyte-specific circHIPK3 knockout mice. (A) Schematic illustration of the breeding strategy to generate inducible cardiomyocyte-specific circHIPK3 knockout (CKO) mice. **(B)** Schematic illustration of the location of primers in genotype identification. F and R primers were designed to prove the correct insertion of loxP site. **(C)** The primer sequence for genotype identification. **(D)** Agarose electrophoresis of PCR product of α-MHC-Cre mouse genotype identification. **(E)** Agarose electrophoresis of PCR product of circHIPK3$^{Flox/Flox}$ mouse genotype identification. **(F)** circHIPK3 level in the hearts of Cre mice (αMHC$^{MerCreMer/Wt}$ mice with tamoxifen treatment) and CKO mice. n = 5. **(G)** Cardiac function of Cre and CKO mice. Data were analyzed by two-tailed Student’s t test. n = 5-6.

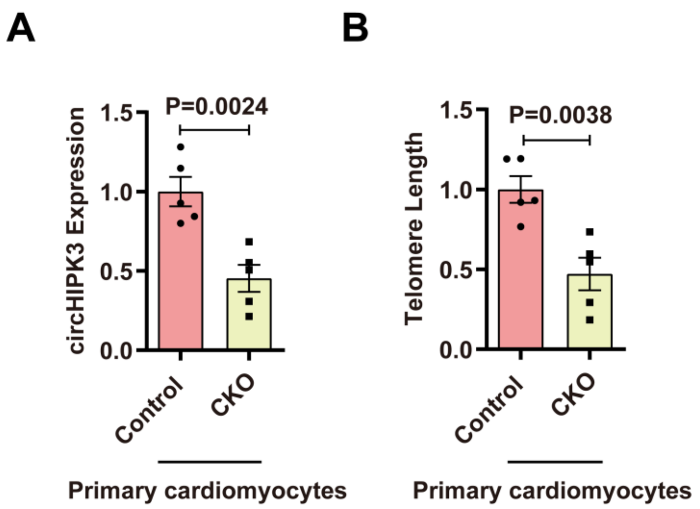

Figure S5 circHIPK3 level and telomere length in isolated primary cardiomyocytes.

(A-B) qRT-PCR analysis of circHIPK3 expression and telomere length in isolated primary cardiomyocytes from control or CKO mice 10 days after tamoxifen injection. n = 5.

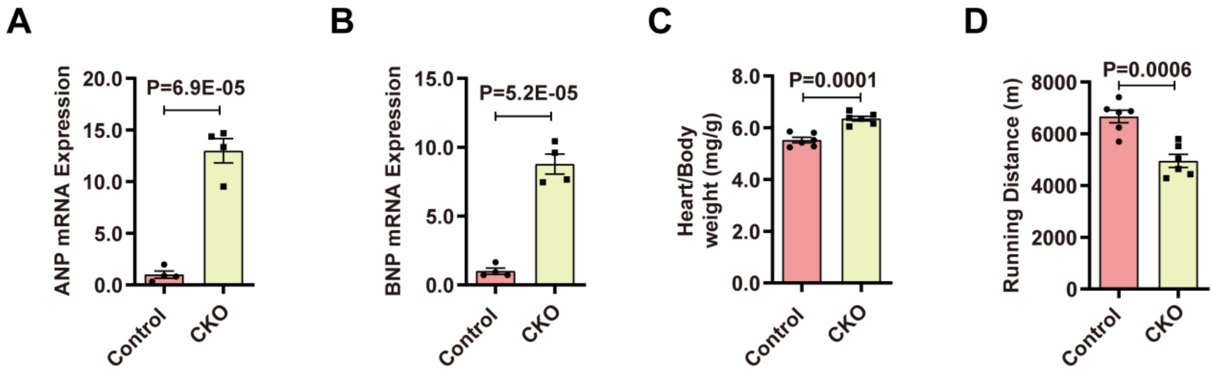

Figure S6 Deletion of circHIPK3 promotes myocardial hypertrophy. (A-B) The expressions of hypertrophy marker ANP and BNP were analyzed by qRT-PCR. n = 4. **(C)** Heart/body weight of CKO and control mice. n = 6. **(D)** Running distance of CKO mice. n = 6. Data were analyzed by two-tailed Student’s t test.

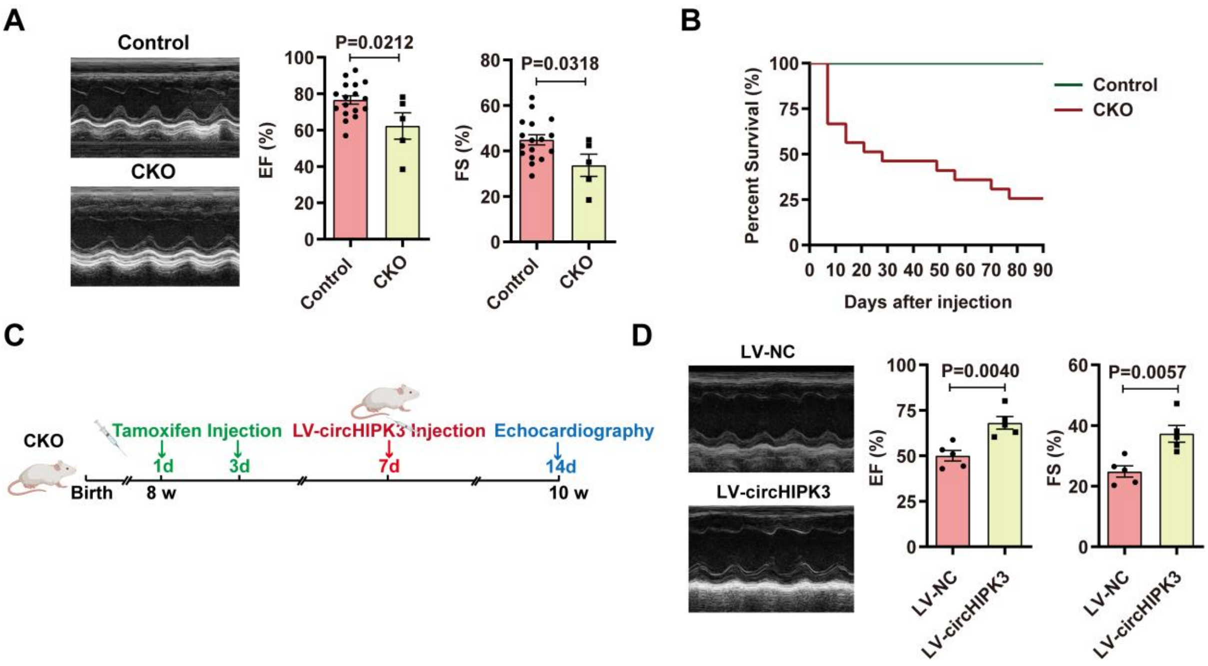

Figure S7 Cardiac function was reduced in the inducible cardiomyocyte-specific circHIPK3 knockout mice after tamoxifen induction. (A) Cardiac function was analyzed by echocardiography for control and circHIPK3 CKO mice 3 months after tamoxifen injection. n = 17 for Control, n = 5 for CKO. **(B)** Survival curve of circHIPK3 CKO mice after tamoxifen injection. n = 17 for Control, n = 18 for CKO. **(C)** Schematics showing that 8-week-old mice were subjected to intraperitoneal injection of tamoxifen at day 1 and 3. At day 7, the mice were infected with a lentivirus infection harboring circHIPK3 via intramyocardial injection. By day 14, the mice were used for subsequent experiment. **(D)** Cardiac function analyzed by echocardiography for LV-NC mice and LV-circHIPK3 mice**.** n = 5. Data were analyzed by two-tailed Student’s t test.

A

Interaction probabilities between circHIPK3 and HuR (human)

CircRNA	Protein	% Identity	Alignment Length	Mismatches	Gap Openings	Tag Start	Tag End	CircRNA Start	CircRNA End
circHIPK3	HuR	100	22	0	0	1	22	1069	1090
circHIPK3	HuR	100	41	0	0	1	41	135	175
circHIPK3	HuR	100	41	0	0	1	41	365	405

B

Interaction probabilities between circHIPK3 and HuR (mouse)

Classifier	Score
Prediction using RF classifier	0.9
Prediction using SVM classifier	0.9

C

Description	Max Score	Total Score	Query Cover	E value	Per. Ident
HuR homology analysis in human and mouse proteins	671	671	100%	0.0	98.16

D

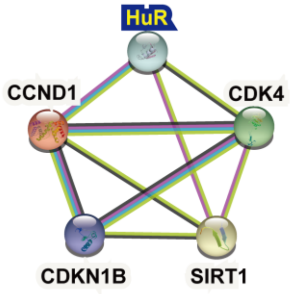

E

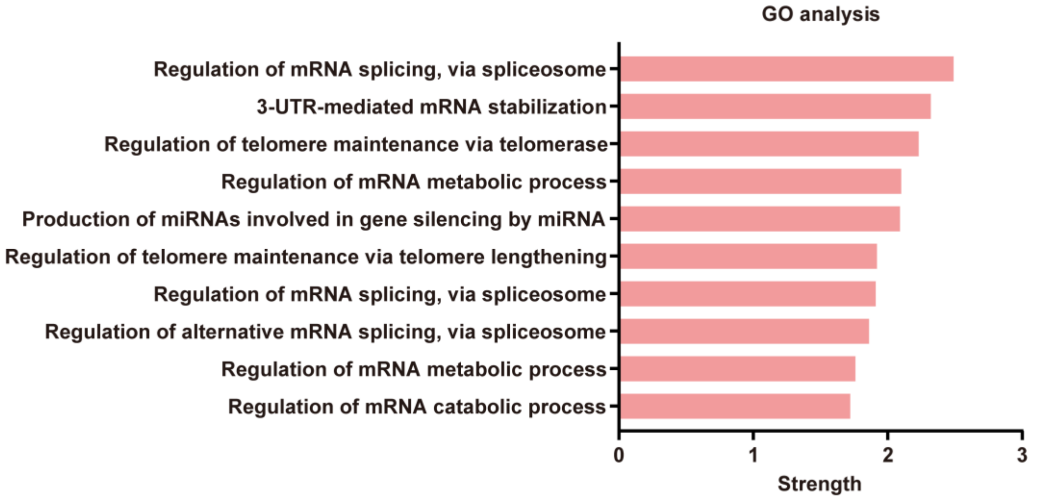

F

Interaction probabilities between circHIPK3 and β-TrCP (mouse)

Classifier	Score
Prediction using RF classifier	0.75
Prediction using SVM classifier	0.98

Figure S8 Prediction of interaction between circHIPK3 and HuR or β-TrCP. (A) Prediction of binding propensities for circHIPK3 and HuR in human by CircInteractome. **(B)** The interaction score for circHIPK3 and HuR in mouse was predicted by RPISeq. **(C)** BLAST alignment showing the conservation between human and mouse. **(D)** The interaction network of HuR protein was analyzed by the STRING database. **(E)** Gene ontology (GO)

analysis of PPI network associated with HuR. **(F)** The interaction score for circHIPK3 and β-TrCP in mouse was predicted by RPISeq.

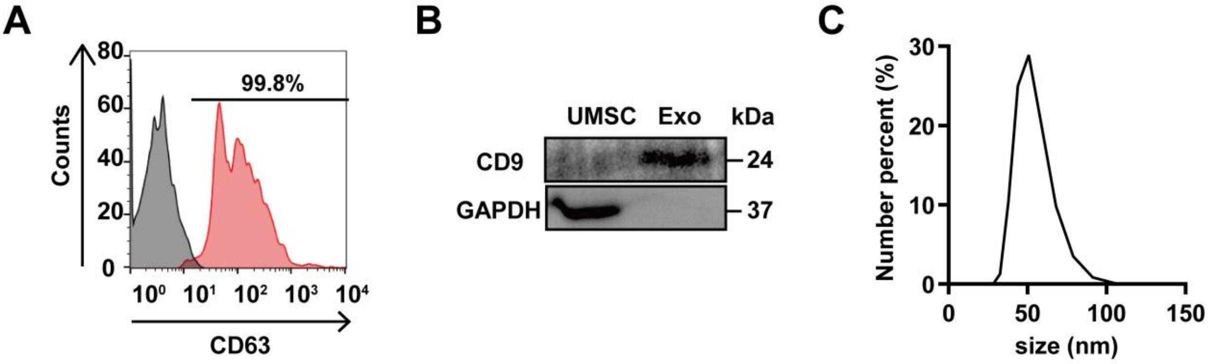

Figure S9 Characterization of exosomes. (A) Flow cytometry analysis of exosomal surface marker CD63. **(B)** The exosomal marker CD9 in UMSC cells and exosomes were analyzed by Western blot. **(C)** Particle size distribution analysis using nanosight tracking analysis. n = 4.

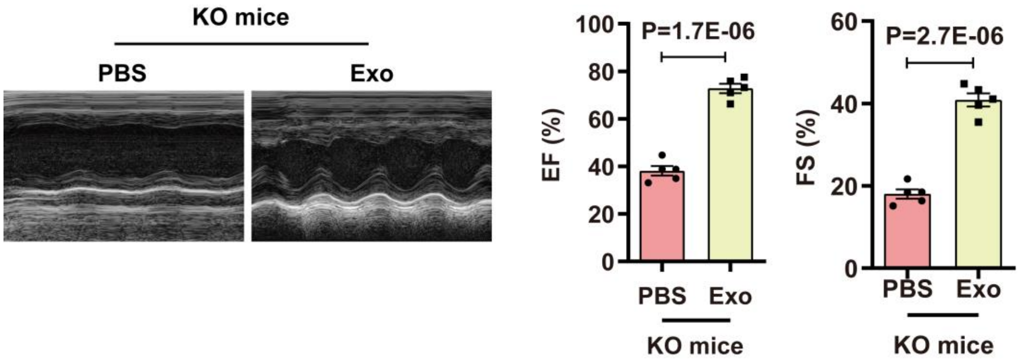

Figure S10 Exosome improved cardiac function in KO mice. PBS or exosome was injected via the tail vein (100 μg) into KO mice three times a week. After four weeks, cardiac function of KO mice was analyzed. n = 5. Data were analyzed by two-tailed Student's t test.

Table S1. The primers used in the RT-PCR assay

Name	Forward primer (5'-3')	Reverse primer (5'-3')
Mouse GAPDH	AAATGGTGAAGGTCGGTGTG	TGAAGGGGTCGTTGATGG
Mouse HuR	GGATGCAACCGACATGTTCAA	AGCGCAGTCTACTTCGGTTT
Mouse p16	CGCAGGTTCTTGGTCACTGT	TGTTCACGAAAGCCAGAGCG
Mouse p21	CCTGGTGATGTCCGACCTG	CCATGAGCGCATCGCAATC
Mouse circHIPK3	GGATCGGCCAGTCATGTATC	ACCGCTTGGCTCTACTTTGA
Rat GAPDH	CAACGGGAAACCCATCACCAT	AGATGATGACCCTTTTGGCCCC
Rat HuR	CTGCTAGGAGGTTTGGAGGC	CGGGGACATTGACACCAGAA
Rat p16	GATAGACTAGCCAGGGCAGC	GAGCTGCCACTTTGACGTTG
Rat p21	GGGATGCATCTATCTTGTGATATGT	AGACGACGGCATACTTTGCT
Rat circHIPK3	GGATCGGCCAGTCATGTATC	ACCGCTTGGCTCTACTTTGA
Mouse 36B4	ACTGGTCTAGGACCCGAGAAG	TCAATGGTGCCTCTGGAGATT
Mouse telomere	CGGTTTGTTTGGGTTTGGGTTTGGGTTT GGGTTTGGGTT	GGCTTGCCTTACCCTTACCCTTACCC TTACCCTTACCCT

Table S2. Sequences of gRNAs

gRNA1	CTATCTTAGCATGAAACTAGTGG	CCACTAGTTTCATGCTAAGATAG
gRNA2	TCTTGGAGCGTTTCAGTGCTTGG	CCAAGCACTCAAACGCTCCAAGA
gRNA3	CGAGACCGAGCCCTATTGTGTGG	CCACACAATAGGGCTCGGTCTCG

Table S3. RNA pulldown probes for circHIPK3

Probe	Sequence (5'-3')
circHIPK3	5bio-ATACCTGTAGTAGCGAGATT
	5bio-CCATACCTGTAGTAGCGAGA
	5bio-AGGCCATACCTGTAGTAGCG
	5bio-TGAGGCCATACCTGTAGTAG
	5bio-TGTGAGGCCATACCTGTAGT
